# Evaluation of a Partial Ban on Rx‐Rebates in Germany

**DOI:** 10.1002/hec.70112

**Published:** 2026-05-18

**Authors:** Maximilian M. Gail, Georg Götz, Daniel Herold, Jan T. Schäfer

**Affiliations:** ^1^ Department of Economics Chair for Industrial Organization Regulation and Antitrust Justus Liebig University Giessen. Licher Strasse 62 Giessen Germany

## Abstract

We investigate patients' price sensitivity for prescription (Rx) drugs with regards to patronizing online or brick‐and‐mortar pharmacies. In doing so, we exploit a policy change in Germany that prohibited online pharmacies from granting rebates to one part of the population, the members of the statutory health insurance scheme. This policy change created a natural experiment, allowing us to analyze its impact on the pharmaceutical market using Difference‐in‐Differences (DiD). Utilizing a novel dataset obtained from merchandize information systems, we find that the ban led to a shift in consumer behavior, increasing offline pharmacy Rx sales by 1.42%–1.67%. In a second step, we assess to what extent the policy change achieved its alleged goal of supporting brick‐and‐mortar pharmacies. Our findings indicate that pharmacies with low revenues, which are most exposed to market exit, saw only a minor increase in annual revenues of around € 1587. At the same time, pharmacies in the highest decile gained roughly four times that amount. This indicates that the policy change alone was insufficient to reverse the declining trend in pharmacy numbers in Germany. To strengthen the comprehensive supply of pharmaceuticals to the general population, additional reforms seem necessary.

## 1 Introduction

The accessibility of pharmaceutical products stands as a cornerstone of public health, directly influencing treatment availability and adherence (Mays et al. 2009; Herwartz and Schley 2018; Haschka et al. 2020; Li and Liu 2021; Atella et al. 2017). This availability is shaped by price and non‐price factors of medications, such as the spatial density of brick‐and‐mortar pharmacies. The emergence and rapid growth of online pharmacies have introduced a significant shift in this landscape, offering consumers potentially lower prices, particularly for over‐the‐counter (OTC), and, in some countries, also for prescription (Rx) drugs, and increased convenience (Lostakova et al. 2012; Heinsohn and Flessa 2013; Long et al. 2022). This enhanced accessibility, however, comes with potential trade‐offs for the traditional brick‐and‐mortar pharmacy sector.

The increasing competition from online retailers poses a challenge to the viability of offline pharmacies, potentially leading to closures as consumers seek lower online prices. This is a familiar pattern from other industries (An and Chung 2023; Chava et al. 2024), and raises concerns about the geographic availability of physical pharmacies. A dense network of those pharmacies is shown to be vital for providing not only medications but also essential health advice and immediate pharmaceutical support (Di Novi et al. 2020; Catalano et al. 2024). The emergence of “pharmacy deserts” could disproportionately affect vulnerable populations, those with limited digital literacy, and individuals needing immediate access. Increasing the density of the pharmacy network, however, usually comes at a cost because, for example, pharmacy reimbursement would have to increase.

In this environment, regulatory bodies face the challenge of balancing the benefits of lower prices offered by online pharmacies with the need to maintain a robust and geographically diverse network of offline pharmacies.^1^ One key regulatory lever involves interventions that influence drug prices or patient's co‐payments across different sales channels. Understanding how consumers respond to payment differentials between online and offline pharmacies is therefore paramount for policymakers. By analyzing consumer behavior in the face of varying payments, this research aims to provide valuable insights into the potential consequences of different regulatory approaches and their impact on both market dynamics and public health outcomes.

Our paper uses the German market as an example. The German Pharmacy Act requires brick‐and‐mortar pharmacies to provide the general population with access to medications. However, the number of pharmacies dropped by roughly 12.5% from 2010 (21,441) to 2020 (18,753) (ABDA 2024, 9). This decline coincides with a rise in competition from foreign online pharmacies. Their market share for OTC drugs increased from around 5% in 2008 to 20% by 2020 (ABDA 2021; Statista 2024). Initially, regulation rendered prices for Rx drugs uniform, which limited price competition to OTC medications. However, a 2016 European Court of Justice ruling (Case No.: C‐148/15) allowed online pharmacies to also offer discounts in the form of vouchers on Rx drugs (Albrecht et al. 2020). These vouchers indirectly reduced patients' co‐payments. In this ruling, the judges claimed that the German government had failed to show that a system of uniform Rx prices was an effective tool to achieve the alleged goal of securing a comprehensive supply of pharmaceuticals to the general population.

In the light of these developments, on December 15, 2020, the German government implemented the so‐called Local Pharmacy Support Act (“Vor‐Ort‐Apotheken Stärkungsgesetz”, henceforth VOASG), with the goal to strengthen brick‐and‐mortar pharmacies. This law prohibits online pharmacies from rewarding the majority of patients (those covered by the mandatory statutory health insurance scheme, making up roughly 90% of the population) with vouchers for purchasing prescription drugs. The other part of the population, privately insured individuals and self‐pay patients, are still allowed to be granted vouchers. This law, by creating a differential impact on patients with statutory versus private insurance and self‐pay, provides a natural experiment to study the effect of a ban on rebates on prescription drug sales.

We employ a novel dataset for this study, constructed from high‐frequency sales data provided by the major merchandise information system (MIS) suppliers in Germany. This dataset encompasses individual transaction data from approximately 9231 offline pharmacies, representing nearly half of all German pharmacies, for the period January 1, 2018, to October 31, 2022.

We find that rebates in the form of vouchers significantly affect patient's choice regarding online and offline pharmacies: the partial ban led to an increase in offline sales of around 1.42%–1.67% for an average brick‐and‐mortar pharmacy compared to a counterfactual scenario without the ban. This result shows that patients' choice regarding the two retail channels is affected by price differentials. This insight is particularly relevant in light of the 2016 European Court of Justice ruling mentioned above, which legalized vouchers granted by foreign online pharmacies. Our research provides empirical evidence regarding consumers' price‐sensitivity, and therefore, suggesting that online rebates could potentially erode offline sales and profitability.

We further investigate whether the introduction of the VOASG successfully mitigated large‐scale pharmacy closures. Economic theory posits that market exit occurs when opportunity costs exceed revenues, resulting in negative economic profits. Given that pharmacies with lower revenues are generally more susceptible to market exit, we employed a quantile analysis by grouping pharmacies into revenue deciles to assess how the average policy‐induced profits varied across these pharmacies with different revenues. We show that, in absolute terms, large pharmacies benefited more strongly from the rebate ban than smaller pharmacies. For instance, the estimated additional *annual* profits generated by the rebate ban for pharmacies in the first and tenth deciles were approximately €1587 and €6,077, respectively. Notably, the *additional annual profit* for the lowest decile equates to around 40% of the average *monthly* income of an employee in Germany in 2021 (Destatis, https://t.ly/N2dJq). This aspect is particularly relevant because German law requires that pharmacies are owned and run by the pharmacists themselves (owner‐management). The findings also indicate that the majority of pharmacies experienced only a mild increase in profits, with a median increase in profits of €3246. Given these relatively modest gains, it seems unlikely that the policy had a substantial impact on pharmacies' decision to enter or exit the market. This is corroborated by the developments in 2021–2024, when the number of pharmacies dropped from 18,461 (2021) to 17,041 (2024) by another 7.7% of pharmacies closed (ABDA 2024, 2025bib_abda_2025).

This article complements the growing body of research on public health service provision. While many studies have focused on hospitals or physicians in specific countries such as the US (Mays et al. 2009; Duminy et al. 2022), China (Li and Liu 2021), and Germany (Herwartz and Schley 2018; Haschka et al. 2020), our research examines the role of pharmacies. These establishments often serve as the final link in the pharmaceutical supply chain, delivering medications to the general public. Previous research on pharmacies has investigated specific services, such as their role in delivering primary care, improving adherence or providing non‐prescription medications (Smith 2009; Agomo 2012; Perraudin et al. 2016; Costa et al. 2019; Di Novi et al. 2020). Our research, in contrast, analyzes how price competition with online pharmacies affects pharmacy profitability and, consequently, the financial sustainability of their services.

We also contribute to the literature on digitization, particularly the debate surrounding the substitutability of offline and online services (Brynjolfsson and Smith 2000; Brown and Goolsbee 2002; Sinai and Waldfogel 2004; Jin and Kato 2007; Goldmanis et al. 2010; Cavallo 2017; Couture et al. 2021) and “digital public health”, which explores how digitization can improve population health (Iyamu et al. 2022; Wong et al. 2022; Yurkovich et al. 2024). In that respect, we investigate the extent to which online pharmacies complement or substitute traditional brick‐and‐mortar pharmacies in supplying pharmaceuticals to the public (Coenen et al. 2011; an der Heiden and Meyrahn 2017). Given that patients are price sensitive, policymakers should take into account that price competition between the offline and online channel can have a detrimental effect on the network of brick‐and‐mortar pharmacies.

The remainder of this paper is structured as follows. Section 2 provides essential background on Germany's health insurance system, focusing on patient reimbursement, co‐payments, pharmacy remuneration, and the role of online pharmacy vouchers. We then present our data and descriptive statistics in Section 3. Our estimation strategy is detailed in Section 4, with the results of our analysis following in Section 5. Finally, Section 6 discusses the limitations of our study, and Section 7 provides concluding remarks.

## 2 Institutional Background

### 2.1 Patient Reimbursement and Co‐Payments for Rx Drugs in Germany

In Germany, most residents are mandated to hold health insurance.^2^ This system offers two primary options: statutory health insurance and private health insurance. As of 2021, around 73.3 Mio. and 8.7 Mio. Citizens were members of the statutory and private health insurance scheme, respectively.^3^ The statutory health insurance is mandatory for all employees below a certain income threshold.^4^ Employees whose income exceeds this threshold may opt out of the statutory health insurance and enroll in private health insurance instead. The same applies to civil servants and self‐employed individuals, who are not subject to the threshold. However, individuals who are privately insured can only switch back to the statutory health insurance system if their income falls below that income threshold and they are under the age of 55.^5^ Additionally, private health insurers typically conduct a comprehensive health screening of applicants before offering a contract, which allows them to adjust premiums or deny coverage based on pre‐existing conditions. Consequently, privately insured individuals are, on average, wealthier and in better health (see, e.g. (Ochmann et al. 2020)).

Rx drugs are prescribed by physicians and dispensed by both traditional and online pharmacies. This holds irrespective of the patients' insurance. Reimbursement, however, differs between statutory and private health insurance. Members of the statutory health insurance are typically required to make co‐payments, contributing a portion of the drug's cost. These co‐payments are calculated based on the retail price, the pharmacy selling price.^6^ If the pharmacy selling price is …

- …below EUR 5 the co‐payment equals the pharmacy selling price,
- …between EUR 5 and EUR 50 the co‐payment equals EUR 5,
- …between EUR 50 and EUR 100 the co‐payment equals 10% of the pharmacy selling price,
- …above EUR 100 the co‐payment is capped at EUR 10.

Further payments beyond the aforementioned co‐payments are possible, which depend on rebates between the patients' insurance company and the drug manufacturers.^7^ The remaining difference between the price of a prescribed drug and the co‐payment and any applicable rebates is typically covered by the insurance provider.

Privately insured individuals can select from a range of insurance contracts, each offering distinct reimbursement structures. Typically, these schemes require patients to initially cover costs out‐of‐pocket, followed by reimbursement from the insurance provider upon submission of invoices. Contracts can be customized to accommodate individual needs, such as making exceptions for high‐cost hospitalizations.

### 2.2 Pharmacy Remuneration for Rx Drugs

The remuneration for the dispensation of Rx drugs in Germany is regulated. Brick‐and‐mortar and mail‐order pharmacies (including those located abroad), receive a fixed fee of € 8.35 per package, in addition to a variable component that constitutes 3% of the wholesale price. Over time, several components have been introduced which either increase or decrease remuneration. These components and, as a prerequisite for the analyses presented in Section 5.2, a detailed description on how to compute a pharmacy's remuneration for a given prescription, are presented in the Appendix (Section A.2).

### 2.3 Online Pharmacy Vouchers

Patients ordering their prescribed Rx drugs from online pharmacies were rewarded with vouchers. Prior to the introduction of the VOASG, basically all prescriptions were eligible. Since the introduction of the law, online pharmacies are no longer allowed to give vouchers for prescriptions that are covered by the statutory health insurance. These vouchers typically offer a value ranging from €2.50 to €10. They are redeemable against the purchase of non‐prescription products or can be credited to the customer's account.^8^

In economic terms, these vouchers function similarly to a rebate on Rx drugs, with the distinction that their application is restricted to lowering the price of OTC drugs bought from the specific online pharmacy. Given the typically low price elasticity of demand for Rx drugs (see Section 4.1), such a rebate primarily represents a transfer from the pharmacy to the patient, thereby functioning as a mechanism for price competition. However, it is crucial to consider that patients' out‐of‐pocket payments are only a fraction of the total cost of an Rx drug—the co‐payments (see Section 2.1). Moreover, a stated policy objective is to ensure a safe and high‐quality supply by pharmacies of medicinal products throughout the country.^9^ That goal might be compromised due to vouchers and the resulting shift in sales away from local pharmacies. In that case, insurance premiums might increase to maintain the minimum standard of services. Consequently, interpreting rebates on Rx drugs in the same manner as rebates on conventional goods is inappropriate. Further discussions on the distributional dimension of the partial ban on vouchers can be found in Section 5.2.

In the context of this goal, it is important to note that price competition—including practices such as vouchers, rebates or even promotional gifts^10^—is prohibited among German pharmacies. Consequently, the VOASG only impacted mail‐order pharmacies located outside of Germany.^11^

## 3 Data and Descriptive Statistics

### 3.1 Data Handling

Our analyses are based on three data sets. First, and central to the analyses, is a unique data set that comprises information on sales of German brick‐and‐mortar pharmacies. The data were obtained from three major suppliers of MIS: AWINTA, ADG and Pharmatechnik.^12^ MIS oversee the entire system of inventory management and provide both hardware and software solutions to pharmacies, essentially handling the IT infrastructure. Our data includes all transactions conducted by a pharmacist with a customer. Only specialty drugs (e.g., cytostatics) are excluded. The data also contain information on pharmacy selling price, transaction revenue, patient co‐payments, and the central pharmaceutical number (PCN) that uniquely identifies each product. To ensure customer anonymity and protect trade secrets, pharmacy location information is limited to the first two digits of the zip code. In Germany, there are 95 two‐digit zip code regions.

The MIS dataset encompasses transactions of 9231 brick‐and‐mortar pharmacies, covering nearly half of all pharmacies in Germany. The data capture individual sales transactions from January 1, 2018, to October 31, 2022. As a result of data preparation (in particular, balancing the MIS data and merging them with the following two data sets), the number of observations in the actual analyses is lower, as will be explained below.

The second data set contains information on package sizes or doses, which are classified using the N‐classification system established by the Federal Institute for Drugs and Medical Devices (BfArM) in Germany.^13^ The N‐classification system categorizes pharmaceutical packages into three size classes based on estimated daily doses: N1 (approximately 10 days), N2 (approximately 30 days), and N3 (approximately 100 days).^14^ Data on N‐classification for each PCN were obtained from the healthcare data and analytics provider IQVIA and from the largest German health insurance fund *Techniker Krankenkasse*.^15^

Data on package sizes is used for three reasons. First, package sizes are used as controls in our estimations (see Section 4.2). Second, to ensure our analyses are restricted to pharmaceuticals, we excluded all items from the MIS dataset lacking package size information, that is, medical devices such as prescribed face‐masks, anti‐thrombosis stockings and ventilators, or other special items such as Covid‐19 vaccines. Third, a robustness check is performed where sales are measured in terms of doses instead of packages (see Appendix, Section A.6).

The third data set comprises information on the evolution of health insurance memberships. The data are obtained from the Federal Ministry of Health and the Association of Private Health Insurance.^16^

As a first step of this process of preparing the data, we balanced the MIS data set. To ensure a consistent sample, we excluded pharmacies that were not continuously active throughout the entire observation period. Pharmacies could have exited the sample due to closure or inactivity, a change in legal name, or a switch in their MIS provider. We also excluded pharmacies with minimal sales and those specializing in the supply of expensive medications. To mitigate volatility in prescription data between groups, which can, for instance, be attributed to seasonal patterns (e.g., influenza waves) and the effects of the COVID‐19 pandemic (e.g., lockdowns, infection waves), we aggregated the data to an annual level. We excluded 2022 from the analysis, as the data set covers only the first three quarters of that year. As a second step, and as explained above, we used data on N‐classifications to ensure that the data set only contains sales of actual pharmaceuticals.^17^ Our data set ranges from 2018 to 2021 and contains information on 6396 pharmacies. This represents around 35% of the total number of pharmacies in Germany over the course of our sample. The estimations presented below are either conducted on the two‐digit zip code‐level or on the pharmacy‐level, which affects the number of observations.

**FIGURE 1:**
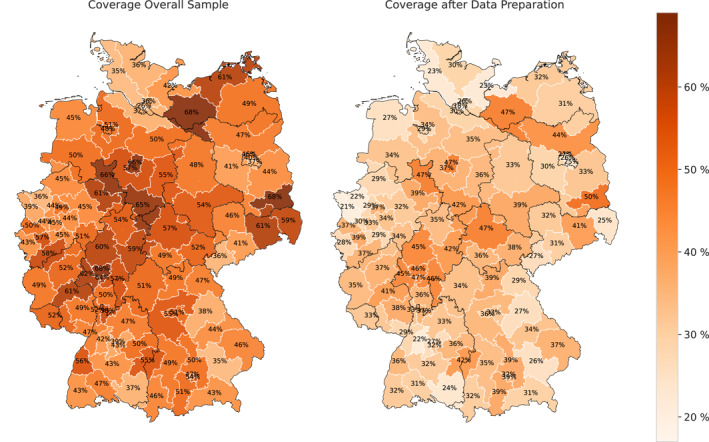
Coverage of Pharmacy Sales Data Across German Regions. *Source:* MIS suppliers and web page of *Apothekenumschau* (https://www.apotheken‐umschau.de/apothekenfinder/), scraped on September 29, 2020.

Figure 1 visualizes the geographic scope of pharmacy sales data across Germany. The left panel illustrates the overall sample coverage, encompassing 9231 pharmacies (roughly 50% of all German pharmacies during the observation period). The right panel zooms in on the coverage of the final sample, which comprises 6396 pharmacies (roughly 35% of all German pharmacies over the observation period). The color intensity within each region corresponds to the percentage of coverage, with darker shades signifying higher data representation. Each two‐digit zip code area is outlined in white, and the borders of Germany's federal states are shown in black. A comparison of both panels reveals that, despite data balancing and processing, our sample retains coverage across all two‐digit zip codes and federal states in Germany, with a median coverage of approximately 34% per two‐digit zip code.

### 3.2 Descriptive Statistics

Table 1 reports summary statistics at the pharmacy level, differentiated between private (Table 1a) and statutory health prescriptions (Table 1b).^18^

**TABLE 1:** Summary statistics on the pharmacy level sample. 51,168 observations in total.

	*N*	Mean	SD	Min	Median	Max
(a) Summary statistics for private prescriptions.						
Sales in packages	25,584	4813	3163	10	4070	44,925
Sales in doses	25,584	298,614	197,482	460	252,505	2,974,320
Gross revenue in Euro	25,584	270,516	306,129	162	210,954	25,378,764
Net revenue in Euro	25,584	228,072	258,416	136	177,681	21,326,693
Net revenue (w.o. Lump sum fees) in Euro	25,584	227,170	258,020	134	176,931	21,320,546
Total remuneration in Euro	25,584	45,634	31,097	85	38,357	851,716
Average remuneration in Euro	25,584	9.44	0.681	8.5	9.32	32.6
Gross pharmacy selling price in Euro/Package	25,584	54.5	27.7	16.2	49.7	999
Net pharmacy selling price in Euro/Package	25,584	45.9	23.4	13.6	41.9	840
Quantity weighted average doses	25,584	62	5.49	14.3	62.7	82.4
(b) Summary statistics for statutory health prescriptions.						
Sales in packages	25,584	29,565	15,673	46	26,350	161,496
Sales in doses	25,584	2,104,195	1,153,345	3200	1,875,830	13,016,770
Gross revenue in Euro	25,584	1,727,631	1,229,671	1094	1,459,565	43,379,709
Net revenue in Euro	25,584	1,456,591	1,037,334	935	1,230,356	36,453,413
Net revenue (w.o. Lump sum fees) in Euros	25,584	1,407,000	1,018,456	853	1,183,201	36,375,761
Total remuneration in Euro	25,584	237,891	127,178	343	211,198	1,362,374
Average remuneration in Euro	25,584	8.07	0.779	7.18	7.92	36.1
Gross pharmacy selling price in Euro/Package	25,584	59.2	31.7	22.8	53.4	1206
Net pharmacy selling price in Euro/Package	25,584	49.9	26.7	19.5	45	1013
Quantity weighted average doses	25,584	70.7	6.05	26.8	71.5	88.7

The descriptive statistics include the number of observations (*N*), mean, standard deviation (SD), median, minimum, and maximum for annual sales in packages or doses, gross revenue, net revenue, net revenue excluding lump sum fees, total remuneration, quantity weighted average doses, average remuneration, and both gross and net pharmacy selling price.

**FIGURE 2:**
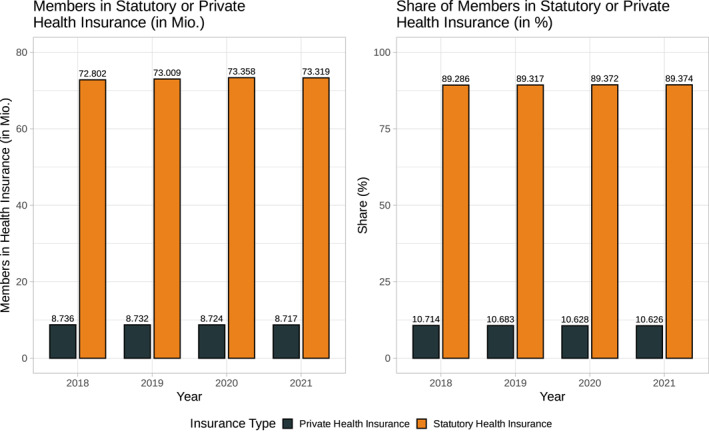
Evolution of private and statutory health insured members.

Figure 2 shows the evolution of memberships in the statutory and private health insurance. It can be observed that membership shares remain stable over the observed period.

## 4 Estimation Strategy

### 4.1 Identification

In this section, we outline our identification strategy, which leverages the differential impact of the VOASG—introduced in December 2020—on members compared to non‐members of the statutory health insurance system. This natural experiment allows us to apply a difference‐in‐differences (DiD) approach and to conduct an event study to assess causal effects. Prescriptions to members of the statutory health insurance, directly affected by the VOASG, form the treatment group, while prescriptions to privately insured individuals and self‐pay patients, unaffected by the reform, serve as the control group.

Using privately insured and self‐pay patients as a control group for the policy analysis at hand is justified because the treatment was explicitly aimed at the statutory health insurance population. The policy change, the introduction of the VOASG, directly impacted the financial incentives for that population by banning rebates on prescription drugs from mail‐order pharmacies. In contrast, the financial incentives for privately insured and self‐pay patients in terms of buying drugs remained unchanged; they could still receive rebates from foreign mail‐order pharmacies. This makes them a valid control group for a DiD analysis, as they were not exposed to the policy intervention.

The subsequent discussion will elaborate on the characteristics of the market and the two insurance groups vis‐à‐vis the stable unit treatment value assumption (SUTVA), anticipation and selection effects as well as the common trends assumption.

As described above, a central feature of Rx drugs is that they are prescribed by physicians and dispensed by both traditional and online pharmacies. This general rule applies to all physicians in Germany and holds irrespective of insurance type. Both the private and the statutory health insurance systems generally adhere to standardized reimbursement rates and co‐payments for prescribed medications. Members of the statutory health insurance cannot easily switch to private health insurance (see below), and opting to pay out‐of‐pocket requires them to cover the full price of the medication, which is much higher than the co‐payment (see Section 2). Irrespective of the specificities of the insurance schemes, drug prices are the same in offline and online channels. The only difference in the price dimension is that, after the VOASG came into effect on December 15, 2020, members of the statutory health insurance were no longer entitled to rebates in the form of vouchers for online purchases. However, these pharmacies are still permitted to provide rebates to privately insured consumers.^19^ We use exactly this policy change in the price dimension as a natural experiment for our analyses. It is also important to note that our analyses are conducted on an annual basis and prescriptions are valid for only 28 days for members of the statutory health insurance and at most 3 months for private prescriptions.^20^ Prescriptions are issued to individual patients and filled independently; the sale of one prescription does not affect the likelihood or volume of other prescriptions being written or dispensed, either within or across pharmacies. These characteristics make interferences or spillovers across units, as well as anticipation and selection effects implausible (for instance, “Ashenfelter dip”; see (Ashenfelter 1978; Heckman and Smith 1999; Roth et al. 2023; Abbring and Van den Berg 2003; Malani and Reif 2015)).

There is also no indication that systematic *level* differences between members of the statutory health insurance and privately insured or self‐pay patients would lead to a violation of the common trend assumption or a lack of overlap in characteristics (common support assumption). Those differences may arise from the specificities of each insurance scheme outlined in Section 2 (type of employment, income thresholds, insurance contracts, and health screening). Rather than systematic level differences, the common trend assumption rests on a parallel evolution over time, which is discussed in this section. Moreover, given the institutional rules and barriers to switching, members of the statutory health insurance could not simply switch to private health insurance (and vice versa, see above), *noncompliance* is not a concern in our analyses. This is corroborated by the observation that the group composition did not change over the observation period (see, Figure 2). Overall, the policy can thus be considered exogenous to the individuals' choice regarding insurance schemes.^21^

To wrap up the discussion on the characteristics of the market and the health insurance schemes in Germany, we briefly discuss the role of co‐payments and rebates from the viewpoint of the patients. The results presented below should be interpreted with the understanding that patients are unlikely to forgo necessary medication solely because of the absence of rebates. The demand for prescription drugs is generally inelastic due to their nature (Gatwood et al. 2014; Yeung et al. 2018): a patient's need for medication is often diagnosed by a physician and is not easily deferred.

As health insurance typically covers the majority of the costs, co‐payments are of minor importance. Furthermore, low‐income patients and those with chronic diseases can be exempt from co‐payments.^22^ Thus, while rebates potentially influence a patient's choice of pharmacy (online or offline), they should not impact overall drug consumption. Given the inelastic nature of demand, any shift in offline sales is expected to be accompanied by a similar shift in online sales. We thus interpret an increase in offline sales as a shift from the online to the offline channel.

Lastly, we quantify the market impact of the VOASG by examining the number of packages dispensed. This metric is of primary importance to our analyses because pharmacy compensation is directly linked to it, and we will use the results of the DiD estimation to assess the additional profits of pharmacies with varying revenue levels.^23^

### 4.2 Static and Dynamic TWFE

Following the distinction in Roth et al. (2023), Chapter 3.2), this section presents the estimation equations of our static and dynamic two‐way fixed effects (TWFE) analyses. The dynamic TWFE, also referred to as event study design, contains leads and lags of the treatment, which allows us to capture dynamic effects and assess pre‐trends.^24^ The estimation equation for the dynamic TWFE model (event study) reads as follows:

(1) $$\ln\left(y_{\mathrm{pgt}}\right)=\alpha_{pg}+\gamma_{t}+\sum_{\tau \neq 2020}\beta_{\tau }D_{g\tau }+W_{\mathrm{pgt}}^{\prime }\mu +\epsilon_{\mathrm{pgt}}.$$

Here, y denotes sales for pharmacies p and statutory health or private prescriptions g at time t (in years). The outcome variable y is measured in logs. Fixed effects $\alpha_{pg}$ and $\gamma_{t}$ are included to capture cross‐sectional heterogeneity for each combination of p and g, as well as time‐varying effects that contemporaneously affected all pharmacies p and prescription types g. The time effects account for common macro‐level shocks, such as national lockdowns during the pandemic. The term $\sum_{\tau \neq 2020}D_{g\tau }$ represents indicator variables that take the value of one if prescription g is a statutory health prescription in the period 2018 to 2021. The model includes 2018–2019 as leads and 2021 as a lag. As is standard in the literature (see Cunningham (2021), Chapter 9.4) or Freyaldenhoven et al. (2019)), the event study is normalized to the last pre‐intervention period (τ=2020), which is therefore excluded from the summation. The coefficient $\beta_{2021}$ in Equation (1) refers to the average treatment effect on the treated (ATT).^25^ The coefficients $\beta_{\tau }\in \left\{2018,2019\right\}$ capture the pre‐treatment effects, which allow us to examine dynamics prior to the policy intervention.

Covariates are included in $W_{\mathrm{pgt}}$ to control for observable differences between pharmacies (or prescription types) whose outcome trends might otherwise diverge. By conditioning on these factors, we strengthen the plausibility that the (conditional) parallel trends assumption holds. In the dynamic TWFE, $W_{\mathrm{pgt}}$ contains the quantity‐weighted average of doses per pharmacy p and group g at time t.^26^ (In the static TWFE, $W_{\mathrm{pgt}}$ contains further covariates, see below.) This covariate is calculated based on Germany's N‐classification system. As explained in Section 3.1, this system categorizes packages into three sizes: N1 (10 doses), N2 (30 doses), and N3 (100 doses). While not exact, this system provides a reasonable approximation of doses per package. To account for potential variations in package sizes that could influence our results, we calculate a weighted average of package sizes at the two‐digit zip code or pharmacy level for each group. For instance, a combination of 15 N1 and 5 N2 packages (a total of 20 packages) would equate to approximately 300 doses. This corresponds to an quantity weighted average of 15 doses per package (300 doses/20 packages). (The appendix (Section A.6) contains a robustness check, where package size instead of number of packages sold is used as the dependent variable.)

Since the outcome variable is measured in logs, $\beta_{\tau }$ is interpreted as a percentage change. The exact ATT reads $e^{\beta_{\tau }}-1$, although this transformation is negligible when the effect is close to zero, as it is in our case.

In the remainder of this section, we will present the static TWFE specification. This is a restricted version of the dynamic TWFE/event study specification in which the treatment is summarized by a single post‐treatment indicator. The static TWFE estimation equation reads

(2) $$\ln\left(y_{\mathrm{pgt}}\right)=\alpha_{pg}+\gamma_{t}+\beta D_{gt}+W_{\mathrm{pgt}}^{\prime }\mu +\epsilon_{\mathrm{pgt}},$$

where the notation is similar as in Equation (1). Similar to the event study, $D_{gt}$ is an indicator variable that equals one if g represents a statutory health prescription at time t=2021. The coefficient β represents the ATT.

Equation (2) is estimated in two variants. First, we include the share of members of the statutory health insurance relative to the total of insured individuals at the national level as a control in $W_{\mathrm{pgt}}$ (see Column (A) in Section 5.1). This covariate is measured at the national level and varies annually. Second, a group‐specific (treatment or control) time trend is included (see Column (B) in Section 5.1). Both variants control for potential confounding effects of shifts in insurance enrollment, even though such effects are unlikely, as indicated by Figure 2.

### 4.3 Heterogeneity Analysis

As explained in Section 1, the VOASG was introduced to strengthen the comprehensive supply of pharmaceuticals to the general population during a period of a shrinking number of brick‐and‐mortar pharmacies. To evaluate the effects of the policy change, we extend the analysis by computing the additional remuneration generated by the introduction of the VOASG on pharmacies with heterogeneous revenues.

Economic theory predicts that, all else equal, pharmacies with lower revenues are more exposed to market exit. We therefore categorize pharmacies into deciles based on their revenue. The first decile includes the 10% of pharmacies with the lowest revenue, the second decile includes the next 10% with the second‐lowest revenue, and so on.

For this analysis, we removed outliers from the dataset to mitigate the influence of potential data errors on the revenue deciles. Given that distributional stratifications are sensitive to extreme observations at the tails, this step ensures more stable subgroup assignments and coefficient estimates. Appendix A.9 presents a comparison of the results.^27^

We tested whether the ATTs differ between pharmacies with different revenues. In doing so, we incorporated an interaction term between the treatment indicator and dummies for the respective revenue deciles into Equation (2). A subsequent Wald test was conducted to evaluate the null hypothesis that the resulting decile‐specific ATT estimates were jointly equal to a single, average ATT (Cameron and Trivedi 2005, Chapter 7.2). This hypothesis could not be rejected. (A detailed discussion is provided in the Appendix, Section A.9.) Based on this finding, we calculate the average additional profits for each pharmacy using the ATT estimate from the TWFE model (2), and present the distribution by decile. The results of this analysis are presented in Section 5.2.

## 5 Results

### 5.1 Static and Dynamic TWFE

Table 2 provides an overview of the estimated coefficients measuring the effect of ban on rebates due to the VOASG (see Equation (1)).^28^ The table contains four different specifications. Column (1) presents results aggregated at the *two‐digit zip code level*. Columns (2) to (4) reports the results for an aggregation at *pharmacy‐level*. The first and second specifications (columns (1) and (2)) respectively contain a fixed effect on the zip code and pharmacy level, each one with robust standard errors.^29^ The third and fourth specifications (columns (3) and (4)) contain a fixed effect at the pharmacy level, with standard errors clustered at the zip code level and at a combination of the zip code and prescription type level, respectively.

**TABLE 2:** Estimation results for Equation (1).

	(1)	(2)	(3)	(4)
Lead 2018	−0.0036	−0.0047^*^	−0.0047^**^	−0.0047
	(0.0034)	(0.0028)	(0.0021)	(0.0034)
Lead 2019	0.0003	0.0017	0.0017	0.0017
	(0.0025)	(0.0020)	(0.0012)	(0.0022)
Lag 2021	0.0161^***^	0.0178^***^	0.0178^***^	0.0178^***^
	(0.0038)	(0.0027)	(0.0015)	(0.0033)
Weighted average of doses	0.0033	−0.0023^*^	−0.0023	−0.0023
	(0.0026)	(0.0014)	(0.0019)	(0.0022)
Observations	760	51,168	51,168	51,168
Adj. R2	0.9997	0.9906	0.9906	0.9906
Fe: Year	X	X	X	X
Fe: 2 digit zip code & prescription type	X			
Fe: Pharmacy & prescription type		X	X	X
Std. Errors	Robust	Robust	By: 2‐Digit‐zip code	By: 2‐Digit‐zip code & prescription type

*Note:* Standard Errors in parenthesis.

^*^ *p* < 0.1.

^**^ *p* < 0.05.

^***^ *p* < 0.01.

**FIGURE 3:**
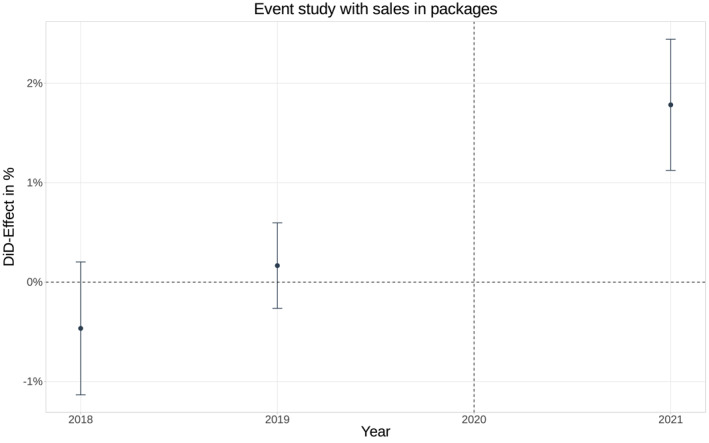
Event study results with 95% confidence intervals based on Table 2 column (4).

In what follows, we use Column (4) as our preferred specification, that is, standard errors clustered at the two‐digit zip code and prescription‐type level. This accounts for spatial correlation in unobserved shocks among pharmacies in the same local market and correlation within prescription‐type groups. The latter is especially relevant because statutory health prescriptions and private prescriptions operate under different institutional regimes (see Section 2). These institutional differences potentially generate correlated shocks, that is demand shocks within each prescription group. The ATT resulting from our preferred specification in the dynamic TWFE estimation reads 0.0178. Figure 3 presents a graphical representation of the event study estimates.

Table 2 shows that the pre‐treatment coefficients $\delta_{2018}$ and $\delta_{2019}$ are statistically insignificant and close to zero in our preferred specification (4). In the alternative specifications (2) and (3), which are not used in the following analyses, the lead for 2018 is significant at the 10%‐level and 5%‐level, respectively. The table also shows that the ATTs are similar across the two aggregation schemes.^30^

**FIGURE A2:**
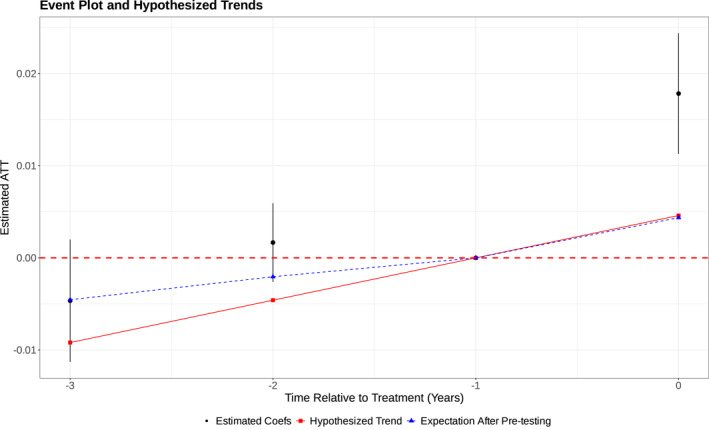
Dynamic DiD Coefficients and Pre‐Test Bias Diagnostics. The plot compares the estimated treatment path (black dots) against the assumed unconditional bias (“Hypothesized Trend”) and the induced selection bias (“Expectation After Pre‐testing”). The large vertical distance between the estimated post‐treatment effect and the bias lines suggests the treatment effect is not driven by confounding trends.

**FIGURE A3:**
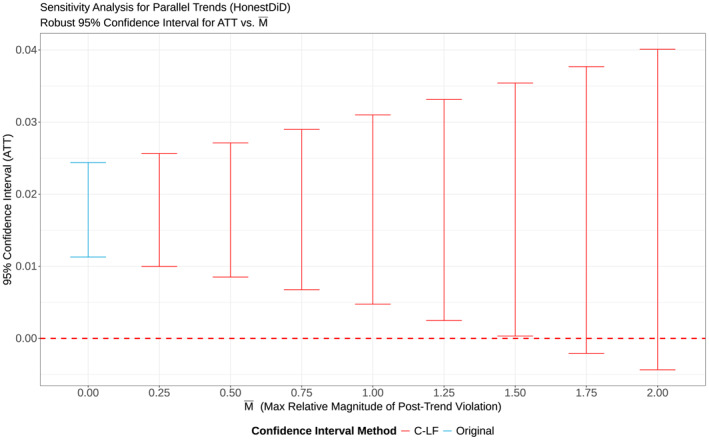
Sensitivity Analysis: Robustness of ATT to Parallel Trends Violation $\left(\bar{M}\right)$. The figure displays the robust 95% confidence interval as a function of the maximum allowed relative bias $\left(\bar{M}\right)$. The ATT is confirmed robust, as the interval remains entirely above zero even when the post‐treatment violation is scaled up to twice the maximum observed pre‐treatment difference.

Overall, the statistical insignificance of the pre‐treatment coefficients provides initial evidence that parallel trends are plausible (Roth et al. 2023, Sections 4.3–4.4). However, to validate this, we conduct sensitivity analyses using the power diagnostic framework of Roth (2022) and the robust inference methods of Rambachan and Roth (2023). The power diagnostics indicate that our results are unlikely to be driven by pre‐testing bias or low statistical power (see Appendix, Figure A2). Furthermore, the robust inference analysis—which allows for valid causal inference even if parallel trends do not hold exactly—demonstrates that the estimated treatment effects remain statistically significant even when allowing for substantial post‐treatment deviations (nearly 1.75 times the magnitude of the maximum observed pre‐treatment difference; see Appendix, Figure A3). These detailed robustness checks are presented in the Appendix, Section A.4.

Table 3 presents the results of the static TWFE model (see Equation (2)).^31^ We estimate two model specifications: a baseline model (A) and a model with a time‐trend (B).

**TABLE 3:** Results of the DiD‐estimation.

	Column A: Without trend	Column B: With trend
ATT	0.0167^***^	0.0142^***^
	(0.0034)	(0.0037)
Share of statutory health insured members	2.2213	
	(1.9185)	
Weighted average of doses	−0.0022	−0.0022
	(0.0021)	(0.0022)
Time trend of treatment‐group		0.0023
		(0.0017)
Observations	51,168	51,168
Adj. R2	0.9906	0.9906
Fe: Year	X	X
Fe: Pharmacy & prescription type	X	X
Std. Errors	By: 2‐Digit‐zip code & prescription type	By: 2‐Digit‐zip code & prescription type

*Note:* Standard Errors in parenthesis.

*p < 0.1.

**p < 0.05.

^***^ *p* < 0.01.

Table 3 shows an ATT of 0.0167 without a time trend (Column A) and 0.0142 with a time trend (Column). All results are statistically significant at the 1% level. Thus, sales in brick‐and‐mortar pharmacies increased by 1.42%–1.67% due the ban on Rx rebates introduced by the VOASG compared to a counterfactual scenario without the policy. This suggests that, even though the *overall demand* for Rx drugs is inelastic (Gatwood et al., 2014; Yeung et al., 2018), a notable portion of patients is price sensitive when it comes to the *choice of the retail channel*. Thus Rx rebates lead to patients switching to that sales channel where Rx drugs are offered more cheaply.^32^

### 5.2 Heterogeneity Analysis

**FIGURE 4:**
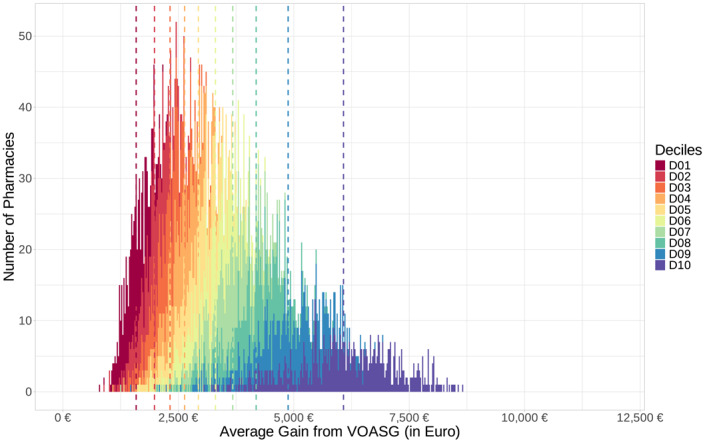
DiD coefficient β=0.0149 from Table 3, Panel B. AverageGainfromVOASG = $Sales\times \frac{\beta }{1+\beta }\times Average\;Remuneration\;Per\;Sale$.

In this section, we compute the average additional profits that pharmacies in each revenue decile realized per year due to the introduction of the VOASG. As explained in Section 4.3, we use the ATT that results from Estimation 2 to quantify the impact of the policy, in particular, the ATT obtained from the static TWFE estimation with a time trend.^33^ The results are presented in Figure 4 and reveal a notable heterogeneity in the magnitude of the effects across different revenue deciles.

Figure 4 illustrates the distribution of the absolute impact of the partial ban on rebates across pharmacies with varying revenue levels. This figure was constructed by multiplying each pharmacy's remuneration for dispensing Rx drugs by the ATT 0.0149.

The dotted lines in Figure 4 represent the average increase in profits for each decile. Our findings suggest that, on average, the rebate ban generated additional annual profits for pharmacies in the first decile of around €1587. In contrast, the corresponding gain for pharmacies in the 10th decile is roughly four times greater, reaching €6077. Figure 4 also indicates that 50% of the pharmacies experienced an increase in profits of less than €3246 (median). In contrast, the average gain across all pharmacies is at €3500. The distribution of the average gains from the policy between deciles is left‐skewed, which indicates that these gains were unevenly distributed across pharmacies, with *larger* pharmacies actually benefiting more strongly.

These results can be further contextualized. As discussed previously, pharmacies exit the market when opportunity costs exceed revenues. The question, therefore, is whether the additional profits are meaningful enough to sustain pharmacies in the market. To evaluate this, consider that the average *monthly* income of a German employee in 2021 was €4100 (Destatis, https://t.ly/N2dJq). In contrast, the *annual* gain for pharmacies in the first decile approximates 40% of this amount. The median pharmacy experienced an increase equivalent to 80% of this figure. In other words, pharmacy owners' yearly gains were substantially less than the average monthly wage of an employee. Given that these owners are highly skilled professionals, the impact of the VOASG on their revenues can be considered relatively low.

Against the backdrop of these findings, the continued decline in the number of brick‐and‐mortar pharmacies after 2020 is unsurprising. By the end of 2024, the total number had decreased to 17,041, reflecting a 9.1% reduction compared to 2020, where that number was 18,753 (ABDA 2025). While external factors, such as the war in Ukraine and the pandemic, influenced the market, the policy change appears to have failed to address the underlying causes of pharmacy closures.

Using the estimated ATT (see Section 5.1), we also computed the foregone savings of consumers due to the ban on rebates. In doing so, we assume that the partial ban on vouchers leads to an increase in expenditure of those patients who, without the policy change, would have purchased online. Given that vouchers historically ranged between €2.5 and €10, the estimated aggregate foregone savings of those prescriptions that are no longer filled online amount to €9.1 million in our sample.

This value should not be directly interpreted as a net loss in consumer surplus for the following reason. The German regulatory framework designates pharmacies as fulfilling a central public health mandate, specifically ensuring a “safe and high‐quality supply of medicinal products to the German population” (ECJ ruling C‐148/15, par. 31). Competition from mail‐order pharmacies potentially erodes the revenue base (rents) necessary for maintaining the required density of the brick‐and‐mortar network. Should the viability of this network be compromised, supplementary funding would be necessary to maintain the minimum standard of service. These additional costs would most likely be drawn from the health insurance system. Accordingly, aggregate patient savings from Rx rebates could potentially be offset by higher insurance premiums necessary to subsidize and sustain the stationary pharmacy infrastructure. The political and distributional dimensions of Rx rebates are beyond the scope of this article.

## 6 Limitations

Our study is subject to certain limitations. While it relies on the most comprehensive and detailed dataset available on the German pharmacy market (to our knowledge), the data is limited to the two‐digit zip code level. Consequently, we are unable to further specify the precise location of a given pharmacy. If the data were more granular, we could have conditioned the effects on specific socio‐geographic factors, such as rural versus urban areas and the specific competitive landscape of offline pharmacies.

A comparable dataset encompassing online sales is currently unavailable. Our interpretation, which posits that e‐commerce experiences losses equivalent to the gains of the offline channel, is thus contingent on the assumption of inelastic demand.

It is essential to consider these limitations when interpreting our findings. However, these caveats do not affect the validity of our identification strategy.

## 7 Conclusion

We find that the partial ban on rebates led to an increase in offline sales of around 1.42%–1.67% for an average brick‐and‐mortar pharmacy compared to a counterfactual scenario in which rebates would not have been banned. Even though the aggregate demand for Rx drugs is inelastic (see, e.g., Gatwood et al. (2014); Yeung et al. (2018)), this finding shows that patients are price‐sensitive when selecting their retail channel for purchasing them. Moreover, with an inelastic total demand for Rx drugs, the increase in offline sales corresponds to a decrease in online sales by the same amount. Our findings reveal that a substantial portion of consumers respond to price differences and rebates for Rx drugs by selecting the more affordable retail channel. This insight is particularly relevant in light of the 2016 European Court of Justice ruling (Case No.: C‐148/15) mentioned above, which legalized rebates offered by foreign online pharmacies. In this ruling, the judges claimed that the German government had failed to show that a system of uniform Rx prices was an effective tool to achieve the alleged goal of securing the comprehensive supply of pharmaceuticals to the general population. Our research provides empirical evidence that consumers are price‐sensitive, suggesting that online rebates could potentially erode offline sales and profitability.

The policy change appears to have had an asymmetric effect on pharmacies. We estimated that annual additional profits ranged from €1587—€6077. Notably, the *additional annual profit* for the lowest decile equates to around 40% of the average *monthly* income of an employee in Germany in 2021. The findings also indicate that the majority of pharmacies experienced only a mild increase in profits, with a median increase in profits of €3246. Pharmacies in the top revenue decile experienced an increase in remuneration that was almost four times stronger than the increase in lowest decile. This suggests that larger pharmacies benefited disproportionately from the rebate ban compared to smaller pharmacies.

These findings suggest that while implemented with the goal of supporting at‐risk pharmacies, the law alone is only one part of a broader policy to mitigate market exit. This is corroborated by the developments in 2021–2023, when another 7.7% of pharmacies closed. This suggests that further reforms are necessary to reverse the trend of declining pharmacy numbers. Such reforms can, for instance, specifically target pharmacy remuneration for dispensing Rx drugs.

## Funding

This research was funded by AVWL. The funding supported researcher positions, but the authors were granted full scientific freedom to conduct their research without any influence on the results or conclusions.

## Conflicts of Interest

The authors declare no conflicts of interest.

## Figures and Tables

**FIGURE A1:**
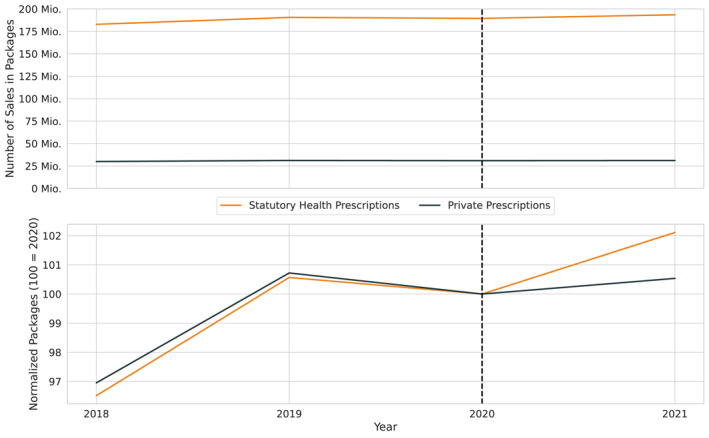
Evolution of sales in packages by statutory health and private prescriptions. The top figure denote sales in absolute values, while the bottom figure depict sales normalized to the year 2020.

**FIGURE A4:**
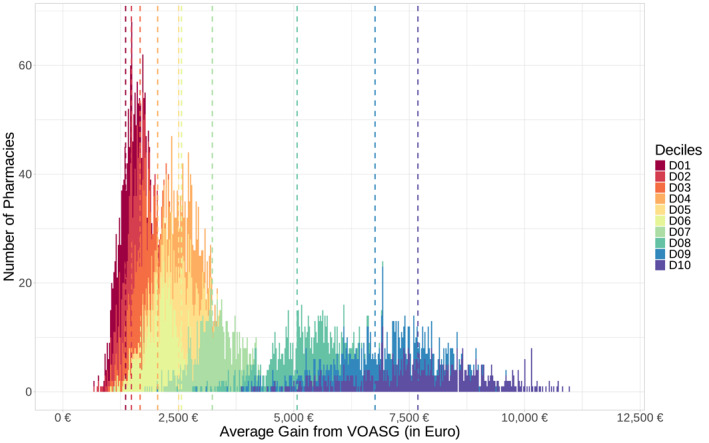
DiD coefficient for each decile (see Table A10). AverageGainfromVOASG = $Sales\times \frac{\beta_{\mathrm{Decile}}}{1+\beta_{\mathrm{Decile}}}\times Average\;Remuneration\;Per\;Sale$.

**FIGURE A5:**
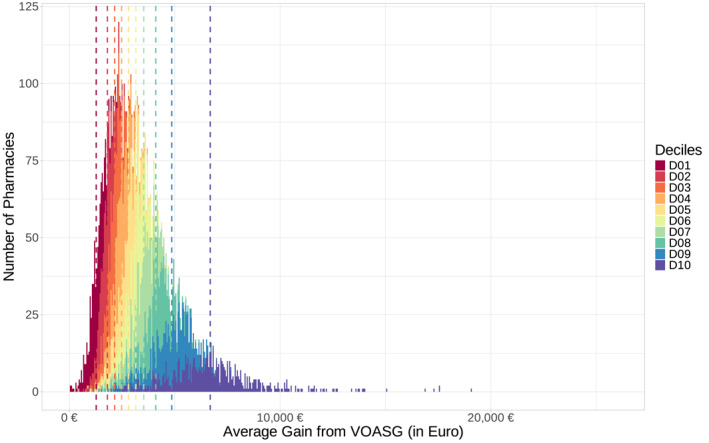
DiD coefficient β=0.0142 from Table 3, Panel B, using the full sample. AverageGainfromVOASG = $Sales\times \frac{\beta }{1+\beta }\times Average\;Remuneration\;Per\;Sale$.

**FIGURE A6:**
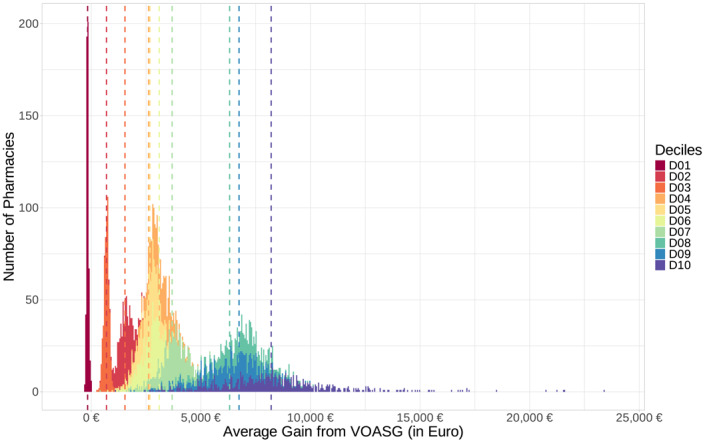
DiD coefficient for each decile (see Table A12) using the full sample. AverageGainfromVOASG = $Sales\times \frac{\beta_{\mathrm{Decile}}}{1+\beta_{\mathrm{Decile}}}\times Average\;Remuneration\;Per\;Sale$.

## Data Availability

The data that support the findings of this study were collected from the commercial MIS providers AWINTA, ADG and Pharmatechnik, the healthcare data and analytics provider IQVIA and the marketing firm Acxiom. Restrictions apply to the availability of these data, which were used for this study.

## Endnotes

^1^
This may include a reliable and safe supply with pharmaceuticals, especially in emergency situations or when pharmaceuticals are difficult to deliver (e.g., due cooling requirements).

^2^
Federal Ministry of Health, https://t.ly/8Ev6L.

^3^
Statista, https://t.ly/pyzwt.

^4^
Further information can be found on the website of the German government: https://www.bva.bund.de/SharedDocs/FAQs/DE/Bundesbedienstete/Arbeit‐Beruf/Entgelt/090jae.html.

^5^
For further information, see, for example, the website of largest German health insurance provider *TK*, https://www.tk.de/vertriebspartner/faq/neuaufnahme/wechsel‐von‐pkv‐zu‐gkv‐2064274.

^6^
A more detailed description of the relevant (and regulated) prices can be found in the Appendix, Section A.2.

^7^
These rebates fluctuate frequently, often on a quarterly basis, and systematic data on their exact amounts is generally unavailable.

^8^
For a detailed description, see the homepage of the two largest online pharmacies active in Germany, *Shopapotheke* and *DocMorris*, https://www.docmorris.de/rezepte/rezept‐bonus and https://www.shop‐apotheke.com/lp/rezeptbonus/. The voucher schemes of the two are fairly similar.

^9^
See paras. 33–34 in the ruling C‐148/15 of the European Court of Justice, https://curia.europa.eu/juris/document/document.jsf?text=&docid=184671&pageIndex=0&doclang=EN&mode=lst&dir=&occ=first&part=1&cid=3568174.

^10^
See, for example, ruling 076/2019 from June 6, 2019 of the German Federal Cout of Justice.

^11^
A small number of German pharmacies also offer mail‐order services. As they were already prohibited from offering rebates, the VOASG did not directly affect their pricing policies, though it did alter the overall competitive landscape.

^12^
See https://www.awinta.de, https://www.adg.de and https://www.pharmatechnik.de.

^13^
Seehttps://www.bfarm.de/DE/Arzneimittel/Arzneimittelinformationen/Packungsgroessen/_node.html.

^14^
An alternative would be to use the concept of daily defined doses (DDD) (see, e.g., WHO Collaborating Centre for Drug Statistics Methodology (2024) for more information). However, the availability of information on DDD is limited in in our data set.

^15^
Seehttps://www.tk.de/resource/blob/2058850/3f65533a18b118a9ebcf585ef2830c40/rabattvertraege‐pzn‐liste‐gesamt‐data.pdf.

^16^
Seehttps://www.bundesgesundheitsministerium.de/themen/krankenversicherung/zahlen‐und‐fakten‐zur‐krankenversicherung/mitglieder‐und‐versicherte and https://www.pkv‐zahlenportal.de/werte/2012/2022/12/pers‐kkv/basket/result.

^17^
To further validate that, in particular, medical devices are excluded from the data set, we used the German pharmaceutical directory *Gelbe Liste Pharmaindex* (https://www.gelbe‐liste.de/).

^18^
Summary statistics at the two‐digit zip code level are reported in Table A1 in the Appendix.

^19^
More information are provided by the German Federal Ministry of Health, see https://www.bundesgesundheitsministerium.de/apotheken.html.

^20^
See https://t1p.de/iu1oo. Yearly aggregation also avoids the risk of confounding treatment effects with timing distortions which could arise from the privately insured delaying filling or bundling prescriptions over weeks.

^21^
We also conducted several sensitivity analyses indicating that the findings are maintained even when allowing for some degree of unobserved post‐treatment violation of parallel trends (see Appendix, Section A.4, esp. Figure A3).

^22^
For further information, see the website of the Federal Ministry of Health, https://www.bundesgesundheitsministerium.de/zuzahlung‐und‐erstattung‐arzneimittel.html.

^23^
Robustness checks using doses instead of packages are reported in the Appendix, Section A.6.

^24^
The estimations are conducted using R's *fixest* package (Bergé 2018).

^25^
The ATT of the dynamic TWFE can be interpreted in a similar way as the ATT of the static TWFE estimation presented below because there is only one post‐treatment period.

^26^
Our results are robust to the inclusion of additional socio‐demographic information (household composition, size and income) as covariates, see the Appendix, Section A.7.

^27^
Appendix A.8 demonstrates that the results of the static and dynamic TWFE estimations are robust to outliers.

^28^
To ensure the robustness of our findings, we performed a simplified DiD analysis by directly comparing the pre‐ and post‐VOASG mean sales between the treatment and control groups. The results of this analysis, which align with those presented in the main text, can be found in the Appendix (Section A.3).

^29^
Note that aggregating the data at the zip code level (column (1)) results in a lower number of observations (95 two‐digit zip code regions observed over four years, differentiated by statutory vs. private prescriptions) than aggregation at the pharmacy level (column (2)), as several pharmacies are located within the same zip code.

^30^
Additionally, the results remain robust when outliers are removed (see Appendix, Tables A7 and A8).

^31^
The Appendix, Section A.5, contains the output table including all four specifications, see above.

^32^
Industry data indicate a 21.5% reduction in reimbursements from statutory health insurance providers to foreign mail‐order pharmacies (ABDA 2023, 19). Considering the estimated small market share of these pharmacies in the prescription drug market (approximately 1% as reported by Albrecht et al. (2020), depending on the basket of pharmaceuticals considered), the estimated ATT appears plausible.

^33^
Note that for the analyses in this section, outliers were removed from the data set (see Section 4.3). The respective ATT for the analysis in this section is 0.0149, which differs slightly from the ATT of 0.0142 reported in Table 3, as shown in Table A7, Panel B, Column (4). Further analyses, including a computation of additional profits using decile specific ATTs and retaining outliers, can be found in the Appendix, Section A.9.

## A Appendix

### A.1 Additional Descriptive Statistics

**TABLE A1:** Summary statistics on the two‐digit‐zip code sample. 760 observations in total.

	*N*	Mean	SD	Min	Median	Max
(a) Summary statistics for private prescriptions.						
Sales in packages	380	324,031	142,870	49,170	318,565	811,735
Sales in doses	380	20,104,566	8,895,330	2,943,970	19,632,930	52,735,480
Gross revenue in Euro	380	18,212,872	8,596,615	2,352,004	17,272,254	47,405,812
Net revenue in Euro	380	15,355,223	7,252,049	1,976,448	14,514,720	39,837,398
Net revenue (w.o. Lump sum fees) in Euro	380	15,294,487	7,225,670	1,965,727	14,452,409	39,659,807
Total remuneration in Euro	380	3,072,350	1,361,241	455,879	2,990,969	7,735,853
Average remuneration in Euro	380	9.47	0.201	9.1	9.44	11.6
Gross pharmacy selling price in Euro/Package	380	55.8	8.15	40.8	54.4	142
Net pharmacy selling price in Euro/Package	380	47	6.91	34.3	45.9	120
Number of pharmacies per 2 digit zip‐code	380	67.3	24.9	15	63	140
Quantity weighted average doses	380	61.9	2.88	49.8	62.2	67.9
(b) Summary statistics for statutory health prescriptions.						
Sales in packages	380	1,990,476	768,530	268,410	1,889,334	4,567,226
Sales in doses	380	141,667,669	55,842,314	16,980,600	137,918,450	352,740,100
Gross revenue in Euro	380	116,315,039	45,882,895	32,142,838	112,818,147	302,983,105
Net revenue in Euro	380	98,066,905	38,704,371	27,010,156	95,233,452	254,601,598
Net revenue (w.o. Lump sum fees) in Euro	380	94,728,159	37,466,388	26,546,225	92,013,684	246,803,740
Total remuneration in Euro	380	16,016,291	6,168,609	2,582,257	15,460,563	37,608,010
Average remuneration in Euro	380	8.06	0.237	7.76	8.02	10.1
Gross pharmacy selling price in Euro/Package	380	59.2	9.63	46.9	57.4	142
Net pharmacy selling price in Euro/Package	380	49.9	8.13	39.4	48.4	119
Number of pharmacies per 2 digit zip‐code	380	67.3	24.9	15	63	140
Quantity weighted average doses	380	71.1	3.15	62.6	70.8	79.3

### A.2 Remuneration in Detail

When dispensing an Rx drug, pharmacists receive a fixed fee of € 8.35 per package and a variable fee of 3% of the wholesale price. The latter is referred to as AEP (§3 AMPreisV). For prescriptions covered by statutory health insurance, this remuneration is reduced by a gross lump‐sum fee of € 1.77 (net € 1.49; see §130 (1) SGB V) paid to the insurance company, provided that remuneration is paid within 10 days.

The remuneration of pharmacies for dispensing Rx drugs is regulated and can be computed based on the pharmacy selling price AVP.

(A1)
(Gross)AVP=(1+VAT)⋅(Net)AVP

(A2)
$$\left(Net\right)\;AVP=r_{f}+r_{v}+pDL+nDZ+AEP.$$

As shown in Equation (A1), gross AVP is calculated by multiplying net AVP by the value‐added tax rate, which is 19% in Germany (16% for the first two quarters of 2020). Net AVP, Equation (A2), consists of a fixed rate, *r*_*f*_ = € 8.35, in addition to a variable component, $r_{v}=0.03\cdot AEP$. Recall that AEP refers to the wholesale price. Moreover, AVP includes two lump‐sum fees per prescription drug in net terms: a fee for so‐called pharmaceutical services (*pharmazeutische Dienstleistung* (pDL)) of *pDL* = € 0.20, and a fee for emergency pharmacy services (*Notdienstzuschlag* (nDZ)) of *nDZ* = € 0.20.^34^ Rearranging Equation (A2) gives the wholesale list price AEP as a function of the AVP and the other components of a pharmacy's remuneration:

(A3)
$$AEP=\frac{\left(Net\right)\;AVP-\left(r_{f}+pDL+nDZ\right)}{1.03}.$$

Based on AEP, we can compute the compensation per Rx‐drug dispensed:

(A4)
$$\begin{array}{cc} R\left(AEP\right) & =\underset{\left(Net\right)\;AVP\;\text{w.o.}\;\text{lump}\;\text{sum}\;\text{fees}}{\underbrace{\left(Net\right)\;AVP-pDL-nDZ-STHF}}-AEP\\ & =r_{f}+r_{v}-STHF\\ & =\text{€}\;8.35+0.03\cdot AEP-\text{€}\;1.49. \end{array}$$

Here, *STHF* = € 1.49 denotes the additional net lump‐sum fee per statutory health insurance prescription drug (with STHF=0 for private prescriptions). For example, an Rx‐drug prescribed under statutory health insurance with *AEP* = € 50 results in a remuneration of *R*(50) = € 8.36.

Regarding the technical implementation, our analysis requires aggregating the previously introduced metrics. We first construct these metrics at the granular transaction level (PCN) and then sum them to the two‐digit zip code or pharmacy level p, differentiated by group g, and year t.

We define *Gross Revenue* as *Gross AVP* multiplied by sales and *Net Revenue* as *Net AVP* multiplied by sales. We also calculate a *Net Revenue without lump sum* *fees* by taking the *Net AVP* excluding the pDL, nDZ, and STHF elements, multiplied by sales. On the cost side, *Total Variable Costs* are defined as the *AEP* multiplied by sales. Based on the *AEP* and sales, we derive the *Total Remuneration*. We also calculate the *Average Remuneration per Sale* by dividing the *Total Remuneration* by the corresponding sales volume.

In Tables A1sss and 1, we provide descriptive statistics for these values, differentiated by private and statutory health prescriptions. Finally, Table A2 summarizes the results by decile. The deciles themselves are constructed based on the *Total Net Revenue* (the sum of *Net Revenue* from private and statutory health prescriptions) for each pharmacy p and year t.

**TABLE A2:** Summary statistics for the presented calculations by deciles 1 to 10.

	Deciles	Mean	SD	Min	P25	Median	P75	Max
Gross revenue (in Euro) from statutory health prescriptions by pharmacy in 2021	D01	580,080	156,013	15,254	491,653	603,985	698,144	854,951
	D02	845,066	104,747	375,124	786,548	850,854	917,596	1,075,456
	D03	1,030,760	105,772	649,517	969,128	1,043,482	1,104,922	1,257,101
	D04	1,194,577	112,944	519,110	1,131,587	1,204,685	1,273,092	1,425,791
	D05	1,375,613	129,665	790,864	1,305,537	1,394,115	1,459,279	1,642,521
	D06	1,567,512	149,516	600,022	1,498,459	1,587,123	1,672,184	1,858,956
	D07	1,797,739	162,622	375,326	1,714,875	1,808,712	1,905,814	2,151,529
	D08	2,093,037	191,242	1,330,587	1,991,520	2,107,104	2,213,523	2,512,798
	D09	2,584,094	277,183	1,006,973	2,400,928	2,570,882	2,777,514	3,255,386
	D10	4,300,888	2,459,499	1,356,382	3,215,381	3,669,349	4,499,821	43,379,709
Net revenue (in Euro) from statutory health prescriptions by pharmacy in 2021	D01	487,448	131,100	12,818	413,153	507,532	586,653	718,418
	D02	710,125	88,023	315,225	660,962	714,970	771,032	903,745
	D03	866,161	88,882	545,816	814,387	876,749	928,504	1,056,386
	D04	1,003,823	94,906	436,225	950,918	1,012,339	1,069,819	1,198,048
	D05	1,155,948	108,966	664,572	1,097,087	1,171,471	1,226,262	1,380,185
	D06	1,317,203	125,639	504,220	1,259,211	1,333,716	1,405,173	1,562,156
	D07	1,510,672	136,647	315,400	1,441,071	1,519,767	1,601,474	1,808,006
	D08	1,758,817	160,707	1,118,132	1,673,543	1,770,661	1,860,093	2,111,606
	D09	2,171,473	232,929	846,196	2,017,586	2,160,422	2,334,044	2,735,619
	D10	3,615,027	2,068,273	1,139,815	2,701,990	3,083,480	3,781,260	36,453,413
Net revenue w.o. Lump sum fees (in Euro) from statutory health prescriptions by pharmacy in 2021	D01	467,598	126,278	12,339	395,049	488,781	562,332	689,964
	D02	682,142	84,526	301,853	634,221	685,886	738,775	871,718
	D03	832,989	85,490	523,595	785,060	843,612	892,430	1,020,360
	D04	965,571	91,483	420,673	913,836	974,303	1,030,302	1,166,132
	D05	1,112,873	104,927	646,402	1,055,069	1,128,102	1,180,965	1,347,118
	D06	1,268,841	120,979	492,045	1,213,262	1,281,520	1,352,468	1,507,382
	D07	1,456,848	132,929	310,243	1,389,072	1,463,156	1,544,132	1,736,182
	D08	1,696,339	155,664	1,077,493	1,610,803	1,709,882	1,794,451	2,049,326
	D09	2,098,092	226,555	826,700	1,949,226	2,089,525	2,255,999	2,650,902
	D10	3,518,845	2,063,884	1,132,121	2,605,533	2,986,705	3,686,305	36,375,761
Total remuneration (in Euro) from statutory health prescriptions by pharmacy in 2021	D01	91,027	26,791	811	75,108	92,412	109,882	161,093
	D02	128,985	24,592	34,619	112,844	129,671	146,051	199,690
	D03	153,614	26,194	54,756	136,446	154,370	169,928	232,846
	D04	177,283	28,328	72,882	159,305	177,330	195,997	279,156
	D05	200,387	35,326	72,548	178,379	205,176	222,864	336,177
	D06	225,541	39,135	61,799	201,654	231,378	252,488	351,801
	D07	252,320	43,782	29,144	230,213	256,633	282,355	395,502
	D08	293,036	53,458	111,920	262,928	298,828	326,168	438,986
	D09	347,264	70,089	100,193	304,126	354,230	395,881	548,914
	D10	477,572	159,606	62,925	383,579	464,683	545,987	1,362,374
Total net revenue (in Euro) per pharmacy from Rx in 2021	D01	578,794	137,690	13,900	506,428	610,769	686,318	742,372
	D02	837,992	54,275	743,267	790,458	840,029	887,387	926,583
	D03	1,012,171	47,992	927,058	971,426	1,012,312	1,053,558	1,093,675
	D04	1,170,606	45,439	1,093,754	1,131,899	1,170,290	1,208,243	1,253,679
	D05	1,342,582	49,182	1,254,728	1,301,047	1,342,594	1,386,771	1,425,902
	D06	1,520,760	54,344	1,426,486	1,471,885	1,523,097	1,567,248	1,616,638
	D07	1,736,137	69,247	1,617,787	1,677,458	1,733,688	1,796,548	1,859,943
	D08	2,020,544	101,603	1,860,461	1,931,778	2,014,162	2,102,377	2,210,105
	D09	2,487,262	178,349	2,211,326	2,326,876	2,472,564	2,634,715	2,833,108
	D10	4,146,048	2,454,613	2,834,122	3,083,185	3,512,988	4,332,263	38,040,902
Sales (in packages) of statutory health prescriptions per pharmacy in 2021	D01	11,622	3577	65	9533	11,674	14,079	21,474
	D02	16,383	3470	3754	14,064	16,472	18,747	26,626
	D03	19,421	3730	4693	17,066	19,527	21,780	30,928
	D04	22,395	4052	9103	19,850	22,368	25,128	37,431
	D05	25,220	5089	6234	22,002	25,870	28,368	44,777
	D06	28,314	5616	7127	24,886	29,058	32,118	47,427
	D07	31,513	6372	3019	28,280	32,110	35,812	52,184
	D08	36,579	7767	8910	32,511	37,480	41,223	57,426
	D09	42,964	10,150	11,115	37,122	44,318	49,844	71,714
	D10	56,315	21,206	4497	43,045	55,847	66,855	153,627
Average remuneration per sale (in Euro) from statutory health prescriptions by pharmacy in 2021	D01	7.88	0.345	7.22	7.7	7.83	7.99	12.5
	D02	7.92	0.251	7.47	7.75	7.87	8.02	9.22
	D03	7.95	0.291	7.53	7.79	7.9	8.05	11.7
	D04	7.95	0.232	7.46	7.81	7.92	8.05	9.58
	D05	8	0.34	7.44	7.82	7.92	8.1	11.6
	D06	8.02	0.318	7.42	7.83	7.95	8.11	10.1
	D07	8.08	0.414	7.58	7.86	7.97	8.14	11
	D08	8.09	0.492	7.53	7.86	7.98	8.14	12.6
	D09	8.19	0.537	7.58	7.9	8.01	8.26	12.3
	D10	8.84	2.17	7.62	7.98	8.19	8.77	36.1

### A.3 Simplified DiD Analysis

A simplified DiD analysis can be conducted by comparing mean sales of the treatment and control groups before and after the introduction of the VOASG are compared. Figure A1 depicts annual sales categorized by prescriptions to members of the statutory health insurance system and private prescriptions. The top subfigure depicts absolute values and the bottom one displays sales normalized to 2020.

Figure A1 shows that Post‐VOASG, prescription drug sales in the treatment group increased relative to the control group.^35^ In particular, calculating the ATT by simply comparing the mean values before and after the treatment (without considering any covariates), we find an ATT of roughly 0.0176, that is, sales of brick‐and‐mortar pharmacies increased by roughly 1.76% compared to a counterfactual of a state without VOASG. This result should be viewed as a first indication.

### A.4 Pre‐Trend Sensitivity Analyses

In Table 3, we presented the results of the dynamic TWFE model derived from Equation (1). The initial assessment of the pre‐treatment period (Section 5.1) showed that the individual coefficients for the pre‐treatment leads in 2018 and 2019 for the preferred specification were not statistically distinguishable from zero. This provides evidence that parallel trends are plausible.

Another common approach that goes beyond individual *t*‐tests involves a joint significance test (e.g., a Wald Test/Chi‐Square Test) to assess inference of the pre‐treatment coefficients simultaneously (see, e.g., Cameron and Trivedi 2005, Chapter 7.2; Roth 2022, Section B). In accordance with this approach, we conduct a standard test for joint significance. Recognizing the potential known limitations of individual *t*‐tests and joint significance tests (Roth 2022), we then proceed to a comprehensive sensitivity analysis, applying the pre‐test power diagnostic framework of Roth (2022) and the robust inference framework of Rambachan and Roth (2023) to assess the validity of our analyses of causality.

A Wald test for joint insignificance of the pre‐trends indicates a mixed pattern across specifications (see Table A3). While column (1) does not reject the null hypothesis of joint insignificance (p=0.476), columns (2), (3), and (4) reject the null hypothesis at the 10%, 1%, and 5% significance levels, respectively (p=0.062,p=0,p=0.0124). Specifically, the rejection in the more stringently clustered specifications (Columns 3 and 4) indicates that a violation of the parallel trends assumption, particularly at the pharmacy aggregation level, cannot be ruled out statistically, by just relying on a joint significance test.

**TABLE A3:** Pre‐trend test on joint significance based on results from Table (2).

	Event Studies			
	(1)	(2)	(3)	(4)
*p*‐value (wald Test/Chi‐Square test) ($H_{0}$: Leads = 0)	0.4756	0.062	0*	0.0124

*Note:* “∗” indicates the p‐value is less than 0.0001 and rounds to zero at four decimal places.

When interpreting these findings, it is important to note that these tests (individual *t*‐tests and joint significance tests) may suffer from low power in the context of pre‐testing in DiD analyses (Roth 2022). To overcome this issue, we conduct further analyses proposed by Roth (2022) and Rambachan and Roth (2023).

The first approach is a power diagnostic framework proposed by Roth (2022). This approach diagnoses potential biases arising from pre‐testing by conducting a power calculation to determine the magnitude of a plausible underlying violation (specifically, assuming a linear trend violation) representing the potential unconditional bias. Afterward, the expected value of the coefficients is calculated conditional on the sample successfully passing the pre‐trend test. This reveals the selection bias in the post‐treatment effect estimates, which is introduced because the analysis is restricted only to samples that “pass” the pre‐trend test (i.e., those that exhibit an empirically flat pre‐trend). By comparing the estimated coefficients with the potential unconditional bias and the induced selection bias, we can assess whether the observed treatment effect is likely driven by these confounding factors rather than a true causal impact.

Second, we employ the relative magnitudes bounds $\left(\Delta^{RM}\right)$ approach proposed by Rambachan and Roth (2023). In doing so, we determine the so‐called *breakdown value*—the maximum plausible violation of parallel trends our result can withstand before losing statistical significance. This method defines a sensitivity parameter $\bar{M}$ which assumes the potential unobserved post‐treatment violation (in trend changes) is no more than $\bar{M}$ times the maximum corresponding difference observed in the pre‐treatment period. We then compute robust confidence intervals for the treatment effect under increasing values of $\bar{M}$. If the confidence interval remains statistically significant (i.e., excludes zero) even for relatively large values of $\bar{M}$, it provides strong evidence that our main conclusion is robust to considerable potential violations of the parallel trends assumption.

The results of both analyses are presented for our preferred specification (Table 2, Column 4) in Figure A2 and A.3, respectively.^36^ We first address the results of the power diagnostics tests (Roth 2022) and then the results of the relative magnitudes bounds approach Rambachan and Roth (2023).

Figure A2 displays the estimated event‐study coefficients (black dots) alongside two counterfactual scenarios designed to diagnose pre‐test bias.

The power diagnostics tests (derived from the *pretrends* package) are conducted with a statistical power of roughly 80%. This value is a widely accepted convention in power analysis for determining the minimum detectable effect size (Cohen 1988), and is therefore proposed for pre‐trend analyses to ensure a high probability of detecting a meaningful violation of the common trend assumption (Roth 2022, Section C). According to a power computation conducted with the *pretrends R* package, targeting 80% power allows our pre‐test to detect a hypothetical linear trend violation with a slope of 0.0046.

This hypothetical trend (“Hypothesized Trend”, solid red line in Figure A2) assumes an underlying linear non‐parallel difference in trends (slope of 0.0046). This trend represents an unconditional bias that would contaminate the post‐treatment estimate if the common trends assumption was violated. Furthermore, a coefficient is estimated conditional on a successful passing of the pre‐trend tests (“Expectation After Pre‐testing”, blue dashed line). This line represents the (hypothetical) conditional bias that would be estimated if one only proceeded with the analysis after successfully passing a pre‐trend test (i.e., finding an insignificant pre‐trend).

Figure A2 shows a substantial distance between the post‐treatment coefficient and both the “Hypothesized Trend” (red solid line) and the “Expectation After Pre‐testing” (blue dashed line). This shows that the magnitude of the observed ATT is significantly larger than either the hypothetical unconditional bias or the selection bias induced by pre‐testing. This shows that our main results are unlikely to be driven by pre‐test biases.

To conclude our power diagnostics test, we compute a Bayes Factor (BF). The BF is the ratio of the probability of passing the pre‐test under a hypothesized alternative trend $\left(H_{A}\right)$ relative to passing under the parallel trends assumption $\left(H_{0}\right)$, where $\text{BF}=P\left(\text{Pass}|H_{A}\right)/P\left(\text{Pass}|H_{0}\right)$. In our case, the computed Bayes Factor is 0.2125. Since 0.2125<1, observing a non‐significant pre‐trend provides evidence in favor of the parallel trends assumption $\left(H_{0}\right)$ over the alternative assumption $\left(H_{A}\right)$. This value not only favors the parallel trends assumption $\left(H_{0}\right)$ but is small enough to provide “moderate” evidence for $H_{0}$ over the alternative $H_{A}$ (Jeffreys 1961, Appendix B; Kass and Raftery 1995; Lee and Wagenmakers 2014, Chapter 7.2).^37^

This sensitivity power analysis provides further evidence that the parallel trends assumption holds and indicates that the estimated effect is unlikely to be driven by a potential pre‐existing trend.

In the remainder of this section, the results of the second sensitivity analysis, the relative magnitudes bounds $\left(\Delta^{RM}\right)$ approach, is presented. Figure A3 displays the robust 95% confidence interval derived from the relative magnitudes bounds $\Delta^{RM}$. The figure plots the robust confidence intervals (red bars) for the ATT as a function of the scaling factor $\bar{M}$. The reference point at $\bar{M}=0$ (blue bar) represents the conventional confidence interval, which is calculated under the strict assumption that the parallel trends condition holds exactly.

The robust confidence interval remains above zero for all tested values of the scaling factor $\bar{M}$ up to a breakdown value of 1.75. This indicates the estimated treatment effect is robust: the significant positive finding is maintained even when allowing the unobserved post‐treatment violation of parallel trends to be nearly twice as large $\left(\bar{M}=1.75\right)$ as the maximum pre‐treatment difference observed in the data. In comparison, the analysis of Silverio‐Murillo et al. (2024), Appendix, Figure 2) finds that their results are no longer significant at $\bar{M}=0.2$. Our breakdown point is nearly 10 times larger. In another comparison, Robbiano et al. (2025), Figure 4) identify a breakdown point of $\bar{M}=1.5$ (86% of our breakdown value), which they characterize as robust to “medium” violations of parallel trends. Finally, our findings appear even more robust than one of the key examples provided in the original methodology paper. In their re‐analysis of Benzarti and Carloni (2019); Rambachan and Roth (2023), Figure 5) show that the robust confidence interval for the average post‐treatment effect (${\bar{\tau }}_{\mathrm{post}}$ in the original paper) includes zero when $\bar{M}=1.0$, while the first‐period effect ($\tau_{2009}$ in the original paper) breaks down around $\bar{M}=2.0$. As our results are robust up to $\bar{M}=1.75$, this sensitivity analysis provides strong support for the validity of our findings, demonstrating a level of robustness that meets that of other available benchmark examples in the literature.

### A.5 All Specifications for the Static TWFE

**TABLE A4:** Results of all specifications of the DiD‐estimation (see Section 5.1).

	(1)	(2)	(3)	(4)
Panel A: Sales in packages				
ATT	0.0155***	0.0167***	0.0167***	0.0167***
	(0.0038)	(0.0028)	(0.0015)	(0.0034)
Share of statutory health insured members	1.7573	2.2213	2.2213*	2.2213
	(1.8341)	(1.5346)	(1.1892)	(1.9185)
Weighted average of doses	0.0036	−0.0022	−0.0022	−0.0022
	(0.0026)	(0.0014)	(0.0019)	(0.0021)
Observations	760	51,168	51,168	51,168
Adj. R2	0.9997	0.9906	0.9906	0.9906
Std. Errors	Robust	Robust	By: 2‐Digit‐zip code	By: 2‐digit‐zip code & prescription type
Panel B: Sales in packages with trends				
ATT	0.0137***	0.0142***	0.0142***	0.0142***
	(0.0046)	(0.0035)	(0.0019)	(0.0037)
Weighted average of doses	0.0034	−0.0022	−0.0022	−0.0022
	(0.0026)	(0.0014)	(0.0019)	(0.0022)
Time trend of treatment‐group	0.0018	0.0023*	0.0023**	0.0023
	(0.0017)	(0.0014)	(0.0011)	(0.0017)
Observations	760	51,168	51,168	51,168
Adj. R2	0.9997	0.9906	0.9906	0.9906
Fe: Year	X	X	X	X
Fe: 2 digit zip code & prescription type	X			
Fe: Pharmacy & prescription type		X	X	X
Std. Errors	Robust	Robust	By: 2‐Digit‐zip code	By: 2‐Digit‐zip code & prescription type

*Note:* Standard Errors in parenthesis.

^*^ *p* < 0.1.

^**^ *p* < 0.05.

^***^ *p* < 0.01.

### A.6 Package Size as Dependent Variable

As a robustness check, we use doses as the dependent variable in Equation (2) instead of incorporating them as a covariate only. Recall that information on doses were obtained from the German N‐classification system, see Section 3.1. Table A5 displays the results of this alternative estimation.

**TABLE A5:** TWFE DiD estimation with doses as dependent variable.

	(1)	(2)	(3)	(4)
Panel A: Sales in NDD				
ATT	0.0140***	0.0153***	0.0153***	0.0153***
	(0.0037)	(0.0027)	(0.0015)	(0.0033)
Share of statutory health insured members	−1.6937	−1.0949	−1.0949	−1.0949
	(1.8100)	(1.5322)	(1.1510)	(1.9052)
Weighted average of doses	0.0196***	0.0145***	0.0145***	0.0145***
	(0.0025)	(0.0013)	(0.0018)	(0.0020)
Observations	760	51,168	51,168	51,168
Adj. R2	0.9998	0.9917	0.9917	0.9917
Std. Errors	Robust	Robust	By: 2‐Digit‐zip code	By: 2‐digit‐zip code & prescription type
Panel B: Sales in NDD with trends				
ATT	0.0146***	0.0152***	0.0152***	0.0152***
	(0.0045)	(0.0034)	(0.0019)	(0.0037)
Weighted average of doses	0.0196***	0.0144***	0.0144***	0.0144***
	(0.0025)	(0.0013)	(0.0018)	(0.0020)
Time trend of treatment‐group	−0.0011	−0.0005	−0.0005	−0.0005
	(0.0017)	(0.0014)	(0.0010)	(0.0017)
Observations	760	51,168	51,168	51,168
Adj. R2	0.9998	0.9917	0.9917	0.9917
Fe: Year	X	X	X	X
Fe: 2 digit zip code & prescription type	X			
Fe: Pharmacy & prescription type		X	X	X
Std. Errors	Robust	Robust	By: 2‐Digit‐zip code	By: 2‐Digit‐zip code & prescription type

*Note:* Standard Errors in parenthesis.

^*^ *p* < 0.1.

^**^ *p* < 0.05.

^***^ *p* < 0.01.

The table's interpretation and structure mirror those of Table A4. The ATT reported in Table A5 are comparable to those in the main text, ranging from 0.0140 to 0.0153.

### A.7 Socio‐Demographic Covariates

In this robustness check, we control for changes in household composition and income across two‐digit zip code regions, accounting for variation in the shares of younger and older residents, average household size, and income levels. We use socio‐demographic data provided by the marketing firm Acxiom.^38^ Following Equation (2), $W_{\mathrm{pgt}}$ is augmented with the following covariates: income per person, the share of individuals below age 18, the share above age 65 (with all others as the reference group), and the shares of one‐to four‐person households (households with five or more members serve as the reference group). Results for Equation (2) are presented in Table A6.

The ATTs reported in Table A6 are close to those in the baseline specifications (Table A4), ranging from 0.0142 to 0.0167.

**TABLE A6:** Estimation results from the static TWFE specification based on Equation (2), including socio‐demographic covariates.

	(1)	(2)	(3)	(4)
Panel A: Sales in packages				
ATT	0.0155***	0.0167***	0.0167***	0.0167***
	(0.0036)	(0.0028)	(0.0015)	(0.0033)
Share of statutory health insured members	1.7806	2.2219	2.2219*	2.2219
	(1.8036)	(1.5348)	(1.1952)	(1.8688)
Weighted average of doses	0.0035	−0.0022	−0.0022	−0.0022
	(0.0024)	(0.0014)	(0.0019)	(0.0022)
Observations	760	51,168	51,168	51,168
Adj. R2	0.9997	0.9906	0.9906	0.9906
Std. Errors	Robust	Robust	By: 2‐Digit‐zip code	By: 2‐digit‐zip code & treated
Panel B: Sales in packages with trends				
ATT	0.0136***	0.0142***	0.0142***	0.0142***
	(0.0045)	(0.0035)	(0.0019)	(0.0038)
Weighted average of doses	0.0034	−0.0022	−0.0022	−0.0022
	(0.0025)	(0.0014)	(0.0019)	(0.0022)
Time trend of treatment‐group	0.0018	0.0023*	0.0023**	0.0023
	(0.0016)	(0.0014)	(0.0011)	(0.0016)
Observations	760	51,168	51,168	51,168
Adj. R2	0.9997	0.9906	0.9906	0.9906
Fe: Year	X	X	X	X
Fe: 2 digit zip code & prescription type	X			
Fe: Pharmacy & prescription type		X	X	X

*Note:* Std. Errors Robust by: 2‐digit‐zip code by: 2‐digit‐zip code & Treated.

^*^ *p* < 0.1.

^**^ *p* < 0.05.

^***^ *p* < 0.01.

### A.8 Outlier Influence

As a robustness check, and in order to conduct the heterogeneity analysis (see Section 4.3), we removed outliers from the dataset. This was done to mitigate the influence of potential data errors on the results. This section demonstrates that the results are robust to outliers.

Outliers were removed using a two‐sided trimming procedure. Pharmacies were removed if, in any given year of our observation period (2018–2021), its total annual prescription sales volume and/or its average annual remuneration per sale fell outside the central 95% range (i.e., below the 2.fifth percentile or above the 97.fifth percentile) of the distribution for that respective year. e.g., Fagereng et al. (2020), 122) apply a similar methodology to their data by trimming the “distribution of returns in each year at the top and bottom 0.5%” to drop outlier observations. In general, our results are robust to trimming these outliers; see Appendix, Section A.8. We also excluded sales via courier services and to nursing homes. This trimming reduces the number of pharmacies in our data set from 6396 to 5487.

The results for the static TWFE specification with the trimmed sample (Table A7) confirm the baseline findings (full sample) reported in Table A4.

**TABLE A7:** Estimation results from the static TWFE specification based on Equation (2), where outliers have been trimmed.

	(1)	(2)	(3)	(4)
Panel A: Sales in packages				
ATT	0.0155***	0.0165***	0.0165***	0.0165***
	(0.0039)	(0.0023)	(0.0015)	(0.0032)
Share of statutory health insured members	1.5718	1.1123	1.1123	1.1123
	(1.8638)	(1.2091)	(1.1569)	(1.7127)
Weighted average of doses	0.0013	−0.0006	−0.0006	−0.0006
	(0.0030)	(0.0006)	(0.0009)	(0.0008)
Observations	760	43,896	43,896	43,896
Adj. R2	0.9997	0.9938	0.9938	0.9938
Std. Errors	Robust	Robust	By: 2‐Digit‐zip code	By: 2‐digit‐zip code & prescription type
Panel B: Sales in packages with trends				
ATT	0.0136***	0.0149***	0.0149***	0.0149***
	(0.0048)	(0.0029)	(0.0018)	(0.0036)
Weighted average of doses	0.0012	−0.0006	−0.0006	−0.0006
	(0.0030)	(0.0006)	(0.0009)	(0.0008)
Time trend of treatment‐group	0.0017	0.0013	0.0013	0.0013
	(0.0017)	(0.0011)	(0.0010)	(0.0015)
Observations	760	43,896	43,896	43,896
Adj. R2	0.9997	0.9938	0.9938	0.9938
Fe: Year	X	X	X	X
Fe: 2 digit zip code & prescription type	X			
Fe: Pharmacy & prescription type		X	X	X
Std. Errors	Robust	Robust	By: 2‐Digit‐zip code	By: 2‐Digit‐zip code & prescription type

*Note:* Standard Errors in parenthesis.

^*^ *p* < 0.1.

^**^ *p* < 0.05.

^***^ *p* < 0.01.

Table A8

**TABLE A8:** Estimation results for Equation (1), where outliers have been trimmed.

	(1)	(2)	(3)	(4)
Lead 2018	−0.0035	−0.0026	−0.0026	−0.0026
	(0.0035)	(0.0022)	(0.0021)	(0.0030)
Lead 2019	0.0011	0.0023	0.0023*	0.0023
	(0.0026)	(0.0016)	(0.0012)	(0.0021)
Lag 2021	0.0162***	0.0174***	0.0174***	0.0174***
	(0.0039)	(0.0023)	(0.0014)	(0.0031)
Weighted average of doses	0.0009	−0.0006	−0.0006	−0.0006
	(0.0031)	(0.0006)	(0.0009)	(0.0008)
Observations	760	43,896	43,896	43,896
Adj. R2	0.9997	0.9938	0.9938	0.9938
Fe: Year	X	X	X	X
Fe: 2 digit zip code & prescription type	X			
Fe: Pharmacy & prescription type		X	X	X
Std. Errors	Robust	Robust	by: 2‐digit‐zip code	by: 2‐digit‐zip code & prescription type

*Note:* Standard Errors in parenthesis.

^*^ *p* < 0.1.

^**^ *p* < 0.05.

^***^ *p* < 0.01.

### A.9 Heterogeneity Analysis

This section is organized into three parts. First, we present the descriptive statistics underlying the figure in Section 5.2. Second, results of the decile‐specific ATT analysis are detailed. Third, to highlight the specific influence of outliers, we present the corresponding results using the full sample at the end of this section.

Since the distribution of pharmacy revenues in the full sample exhibits significant skewness, where outliers potentially influence the definition of the deciles disproportionately, we use the trimmed sample as described in Section A.8. By trimming the sample, we mitigate the noise introduced by these extreme values and prevent outliers (and potential data errors) from driving the results of this distributional analysis. Trimming is particularly important at this point, because the decile‐specific analysis is more susceptible to those outliers, so that estimations based on the trimmed data set are more stable.

Section 5.2, particularly Figure 4, provides a histogram visualizing the *Average Gain from VOASG* for each pharmacy. This measure is derived from the ATT β=0.0149 (see Table A7, Panel B, Column (4)), the volume of sales from statutory health prescriptions per pharmacy, and the *Average Remuneration per Sale* per pharmacy for the year 2021 (see Table A2). The *Average Remuneration per Sale* is calculated for each pharmacy by dividing its total remuneration by its total sales (see Section A.2).

To highlight the derivation, for example, consider a pharmacy that processed 24,000 statutory health prescriptions in 2021, and earned an average of 8 Euro per prescription. The VOASG would have resulted in earnings of:

$$\begin{array}{c} 24,000\times \frac{0.0149}{1+0.0149}\times 8\;\text{Euro}=2,819\;\text{Euro}\\ \left(\text{Average Gain from VOASG}=\text{Sales}\times \frac{\beta }{1+\beta }\times \text{Average Remuneration Per Sale}\right). \end{array}$$

Table A9 displays summary statistics for the *Average gain from VOASG* by decile.

**TABLE A9:** Summary statistics for the *Average gain from VOASG* by decile using β=0.0149 from Table A7, Panel B, Column (4).

	Deciles	Mean	SD	Min	P25	Median	P75	Max
Average gain from VOASG (in Euro) by β	D01	1587	258	795	1399	1582	1775	2253
	D02	1984	329	897	1771	2002	2215	2761
	D03	2320	346	1374	2085	2368	2573	3197
	D04	2635	387	1073	2388	2664	2931	3430
	D05	2933	466	1319	2626	3013	3262	3979
	D06	3304	524	1651	2963	3381	3690	4466
	D07	3678	530	1259	3377	3694	4045	4844
	D08	4185	635	2047	3810	4246	4633	5852
	D09	4877	817	1475	4396	4938	5481	6922
	D10	6077	1111	3061	5410	6076	6854	8665

In the remainder of this section, we present the results of the estimations of decile‐specific ATTs and results using the full sample. In doing so, a modified version of Equation (2) is estimated:

(A5)
$$\ln\left(y_{\mathrm{pgt}}\right)=\alpha_{pg}+\gamma_{t}+\gamma_{d}+\sum_{d=1}^{10}\beta_{d}1\left\{Decile_{pt}=d\right\}\times D_{gt}+W_{\mathrm{pgt}}^{\prime }\mu +\epsilon_{\mathrm{pgt}}.$$

In Equation (A5), the index d denotes the revenue decile to which pharmacy p belongs at time t. The term $\gamma_{d}$ represents fixed effects accounting for decile‐specific heterogeneity. The indicator variable $1\;\left\{Decile_{pt}=d\right\}$ equals one if pharmacy p falls into decile d in period t.^39^ The interaction between the DiD indicator variable $D_{gt}$ and the decile indicators identifies decile‐specific treatment effects, $\beta_{d}$. These coefficients therefore capture decile‐specific ATTs which basically capture whether the treatment had a qualitatively different effect on lower‐revenue versus Higher‐revenue pharmacies. The estimation results are presented in Table A10.

**TABLE A10:** Results of the DiD‐estimation for revenue deciles.

	(1)	(2)	(3)
Weighted average of doses	−0.0017***	−0.0017**	−0.0017**
	(0.0005)	(0.0007)	(0.0007)
DiD for decile 1	0.0128***	0.0128**	0.0128**
	(0.0047)	(0.0053)	(0.0056)
DiD for decile 2	0.0111***	0.0111***	0.0111**
	(0.0043)	(0.0038)	(0.0046)
DiD for decile 3	0.0107***	0.0107**	0.0107**
	(0.0040)	(0.0041)	(0.0043)
DiD for decile 4	0.0146***	0.0146***	0.0146***
	(0.0042)	(0.0041)	(0.0044)
DiD for decile 5	0.0127***	0.0127***	0.0127***
	(0.0040)	(0.0034)	(0.0044)
DiD for decile 6	0.0092**	0.0092**	0.0092**
	(0.0037)	(0.0036)	(0.0040)
DiD for decile 7	0.0131***	0.0131***	0.0131***
	(0.0037)	(0.0030)	(0.0036)
DiD for decile 8	0.0182***	0.0182***	0.0182***
	(0.0038)	(0.0030)	(0.0039)
DiD for decile 9	0.0208***	0.0208***	0.0208***
	(0.0037)	(0.0031)	(0.0037)
DiD for decile 10	0.0190***	0.0190***	0.0190***
	(0.0040)	(0.0039)	(0.0047)
Observations	43,896	43,896	43,896
Adj. R2	0.9959	0.9959	0.9959
Fe: Year	X	X	X
Fe: Pharmacy & prescription type	X	X	X
Fe: Deciles	X	X	X
Std. Errors	Robust	By: 2‐Digit‐zip code	By: 2‐Digit‐zip code & prescription type
Chi‐square test ($H_{0}$: DiD coefficient = 0.0149)	0.204	0.107	0.228

*Note:* Standard Errors in parenthesis.

^*^ *p* < 0.1.

^**^ *p* < 0.05.

^***^ *p* < 0.01.

The coefficients for “ATT for Decile x” capture the interaction between the DiD indicator variable and the respective decile, that is, the decile‐specific ATT.

We conducted a Wald Test to test whether all coefficients are significantly different from the ATT obtained in the main text (β=0.0149, see Section 5). The $H_{0}$ (ATT for Decile 1 = ATT for Decile 2 = … = 0.0149) could not be rejected at the usual *p*‐values. This means that, in statistical terms, the decile‐specific ATTs are not so pronounced that we can assume qualitative differences in the treatment effects of the VOASG for each decile. For completeness, Figure A4 illustrates the distribution of the absolute impact of the partial ban on rebates across pharmacies with varying revenue levels.

Assuming decile‐specific ATTs despite the aforementioned results of the Wald‐test reveals a more pronounced difference in the effects across different revenue deciles. Specifically, the increase in Rx sales for deciles one to seven ranges from approximately 0.92%–1.46% across all panels in Table A10. In contrast, the effect for the highest three deciles is one point five to two times larger, ranging from 1.82% to 2.08%.^40^

The figure was constructed by multiplying each pharmacy's remuneration for dispensing Rx drugs by its corresponding ATT (see Table A10). One can see that the results resemble a bimodal distribution. Low‐revenue pharmacies' additional annual gains (in Deciles 1–7) ranged from roughly €700 to €4300. In contrast, the higher deciles (Deciles 8–10) experienced a more pronounced increase. This can also be seen in Table A11, which reports the descriptive statistics of the average annual gains for each decile.

**TABLE A11:** Summary statistics for the *Average gain from VOASG* by decile using $\beta_{d}$ from Table A10.

	Deciles	Mean	SD	Min	P25	Median	P75	Max
Average gain from VOASG (in Euro) by $\beta_{\mathrm{Deciles}}$	D01	1359	221	681	1198	1355	1520	1929
	D02	1484	246	671	1325	1498	1657	2066
	D03	1671	249	990	1502	1706	1854	2303
	D04	2569	377	1046	2328	2598	2858	3345
	D05	2508	398	1128	2245	2576	2789	3401
	D06	2053	326	1026	1841	2101	2293	2775
	D07	3236	467	1108	2971	3249	3558	4261
	D08	5072	770	2481	4618	5147	5615	7094
	D09	6761	1132	2045	6093	6845	7597	9595
	D10	7688	1405	3872	6845	7687	8671	10,962

Even though, the heterogeneity between pharmacies in this analysis is more pronounced than that reported in Section 5.2, the finding is the same: the gains of smaller pharmacies are noticeably lower than that of higher pharmacies. Thus, both analyses imply that the gains from the introduction of VOASG were unevenly distributed across pharmacies, with *larger* pharmacies actually benefiting more strongly.

To conclude this section, we present the results using the full sample, which retains all outliers (Tables A12, A13, A14). Table A12 presents the decile‐specific ATT estimates for the full sample. A comparison with the trimmed sample (Table A10) reveals that including outliers introduces substantial noise into the estimation, particularly at the lower end of the revenue distribution. For instance, the coefficient for Decile 1 becomes negative and statistically insignificant (−0.0018), while Decile 3 drops to near zero (0.0045, insignificant). Conversely, the estimates for the upper deciles remain large and highly significant.

Crucially, the Wald test for the full sample rejects the null hypothesis that the decile‐specific coefficients are jointly equal to the average ATT (β=0.0142) in Columns (2) and (3) (p=0.004 and p=0.011, respectively). By comparison, with the trimmed the Wald test could not reject the null. This shows how outliers exacerbate the heterogeneity of the policy effects.

The economic implications of these outliers are visualized in Figure A5 and A.6, and summarized in Tables A13 and A14. When allowing for decile‐specific effects (Table A14), the influence of the top‐performing pharmacies becomes apparent. The maximum gain in Decile 10 reaches over €25,000, which is more than three times the maxima observed in the trimmed analysis.

Importantly, however, the results as well as their interpretations presented in the main text become even more apparent. The gains of smaller pharmacies become even lower while those of large pharmacies, in particular those in the 10th decile, become even larger. Thus, the results of the heterogeneity analysis remain qualitatively robust to outliers.

**TABLE A12:** Results of the DiD‐estimation for revenue deciles using the full sample.

	(1)	(2)	(3)
Weighted average of doses	−0.0033***	−0.0033*	−0.0033
	(0.0013)	(0.0017)	(0.0020)
DiD for decile 1	−0.0018	−0.0018	−0.0018
	(0.0114)	(0.0106)	(0.0116)
DiD for decile 2	0.0121**	0.0121***	0.0121**
	(0.0051)	(0.0043)	(0.0053)
DiD for decile 3	0.0045	0.0045	0.0045
	(0.0046)	(0.0040)	(0.0044)
DiD for decile 4	0.0178***	0.0178***	0.0178***
	(0.0043)	(0.0038)	(0.0043)
DiD for decile 5	0.0132***	0.0132***	0.0132***
	(0.0042)	(0.0034)	(0.0046)
DiD for decile 6	0.0120***	0.0120***	0.0120***
	(0.0040)	(0.0036)	(0.0040)
DiD for decile 7	0.0148***	0.0148***	0.0148***
	(0.0040)	(0.0031)	(0.0039)
DiD for decile 8	0.0235***	0.0235***	0.0235***
	(0.0043)	(0.0031)	(0.0042)
DiD for decile 9	0.0185***	0.0185***	0.0185***
	(0.0043)	(0.0033)	(0.0042)
DiD for decile 10	0.0175***	0.0175***	0.0175***
	(0.0051)	(0.0045)	(0.0054)
Observations	51,168	51,168	51,168
Adj. R2	0.9930	0.9930	0.9930
Fe: Year	X	X	X
Fe: Pharmacy & prescription type	X	X	X
Fe: Deciles	X	X	X
Std. Errors	Robust	By: 2‐Digit‐zip code	By: 2‐Digit‐zip code & prescription type
Chi‐square test ($H_{0}$: DiD coefficient = 0.0142)	0.032	0.004	0.011

*Note:* Standard Errors in parenthesis.

^*^ *p* < 0.1.

^**^ *p* < 0.05.

^***^ *p* < 0.01.

Figure A5

Figure A6

**TABLE A13:** Summary statistics for the *Average gain from VOASG* by decile using β=0.0142 from Table 3, Panel B, the full sample.

	Deciles	Mean	SD	Min	P25	Median	P75	Max
Average gain from VOASG (in Euro) by β	D01	1274	375	11.3	1051	1293	1538	2254
	D02	1805	344	484	1579	1814	2044	2794
	D03	2149	367	766	1909	2160	2378	3258
	D04	2481	396	1020	2229	2481	2742	3906
	D05	2804	494	1015	2496	2871	3118	4704
	D06	3156	548	865	2822	3238	3533	4923
	D07	3531	613	408	3221	3591	3951	5534
	D08	4100	748	1566	3679	4181	4564	6142
	D09	4859	981	1402	4255	4957	5539	7681
	D10	6682	2233	880	5367	6502	7640	19,063

**TABLE A14:** Summary statistics for the *Average gain from VOASG* by decile using $\beta_{d}$ from Table A12 and the full sample.

	Deciles	Mean	SD	Min	P25	Median	P75	Max
Average gain from VOASG (in Euro) by $\beta_{\mathrm{Deciles}}$	D01	−165	48.5	−292	−199	−167	−136	−1.47
	D02	1538	293	413	1346	1547	1742	2382
	D03	693	118	247	615	696	766	1050
	D04	3102	496	1275	2787	3102	3429	4884
	D05	2614	461	946	2327	2676	2907	4385
	D06	2677	465	734	2394	2747	2997	4176
	D07	3687	640	426	3364	3750	4126	5779
	D08	6742	1230	2575	6049	6875	7504	10,100
	D09	6312	1274	1821	5528	6439	7196	9978
	D10	8208	2743	1082	6593	7987	9384	23,416
